# Probing the Compositional and Structural Effects on the Electrochemical Performance of Na(Mn-Fe-Ni)O_2_ Cathodes in Sodium-Ion Batteries

**DOI:** 10.1002/bte2.20240083

**Published:** 2025-03-22

**Authors:** Samriddhi Saxena, Hari Narayanan Vasavan, Neha Dagar, Karthik Chinnathambi, Velaga Srihari, Asish Kumar Das, Pratiksha Gami, Sonia Deswal, Pradeep Kumar, Himanshu Kumar Poswal, Sunil Kumar

**Affiliations:** 1 Department of Metallurgical Engineering and Materials Science, Indian Institute of Technology Indore, Simrol, Indore, Madhya Pradesh, India; 2 Micron School of Materials Science and Engineering, Boise State University, Boise, Idaho, USA; 3 High Pressure & Synchrotron Radiation Physics Division, Bhabha Atomic Research Centre, Mumbai, Maharashtra, India; 4 School of Physical Sciences, Indian Institute of Technology Mandi, Mandi, Himachal Pradesh, India

**Keywords:** biphasic structure, electrochemical performance, layered oxides, Mn-Fe-Ni system, Na-ion batteries

## Abstract

This study systematically investigates an Mn-Fe-Ni pseudo-ternary system for Na(Mn-Fe-Ni)O_2_ cathodes, focusing on the effects of varying transition metal fractions on structural and electrochemical properties. X-ray diffraction reveals that increasing Mn content induces biphasic behavior. A higher Ni content reduces the c parameter, while higher Mn and Fe concentrations expand the lattice. Average particle size increases with an increase in Mn content and Fe/Ni ratio. NaMn_0.500_Fe_0.125_Ni_0.375_O_2_ delivers a high specific capacity of ~149 mAh g^−1^ in the 2.0–4.0 V range. Galvanostatic charge-discharge and *dQ/dV* versus V curves suggest that a Ni/Fe ratio > 1 enhances specific capacity and lowers voltage polarization in the materials. NaMn_0.500_-Fe_0.250_Ni_0.250_O_2_ demonstrated the best rate performance, exhibiting 85.7% capacity at 1C and 69.7% at 3C, compared to 0.1C, while biphasic NaMn_0.625_Fe_0.125_Ni_0.250_O_2_ (MFN-512) excelled in cyclic stability, retaining 93% of capacity after 100 cycles. The performance of MFN-512 in a full cell configuration was studied with hard carbon as the anode, resulting in a specific capacity of ~92 mAh g^−1^ and a nominal voltage of ~2.9 V at a 0.1C rate, demonstrating its potential in practical applications. Transmission electron microscopy confirmed the biphasic nature of MFN-512, with columnar growth of P2 and O3 phases. Electrochemical impedance spectroscopy revealed that better-performing samples have lower charge transfer resistance. *Operando* Synchrotron XRD reveals reversible phase transformations in MFN-512, driven by its optimized transition metal ratios and phase fraction. This work outlines a systematic approach to optimizing low-cost, high-performance Mn-Fe-Ni layered oxides.

## 1 ∣ Introduction

The rising demand for energy storage systems (ESSs) and the depletion of lithium resources have intensified the search for alternative battery technologies. With nearly a quarter of global lithium reserves projected to be depleted by EV applications by 2050, sodium-ion batteries (SIBs) have emerged as a promising alternative due to their cost-effectiveness and sustainability. However, their widespread adoption is hindered by lower energy density and shorter cycling life compared to lithium-ion batteries, primarily due to the larger size and higher redox potential of sodium ions [1-3]. Enhancing the performance of SIBs depends on developing energy-dense cathode materials that ensure stable sodium-ion storage, as cathodes play a crucial role in determining battery capacity and voltage [4].

Their key components, including sodium (Na), manganese (Mn), and iron (Fe), are abundantly available in the Earth's crust, making SIBs a more affordable option for large-scale energy storage.

Various cathode materials are being explored for SIBs, with layered transition metal oxides showing promise. They have the basic formula Na_*x*_TMO_2_ (where *x* typically ranges from 0.5 to 1.0, and TM represents transition metals like Mn, Fe, Ni, Cu, etc.). Their key components, including Na, Mn, and Fe, are abundantly available in the Earth's crust, making them a cost-effective option for large-scale energy storage [5, 6]. These materials exhibit decent specific capacities (~130–140 mAh g^−1^) and are relatively easier to synthesize, giving them an edge over other cathode materials [7, 8]. Their crystal structures are categorized into polymorphs such as P2, P3, and O3, defined by the environment of sodium ions (prismatic or octahedral) and the number of oxygen layers [9, 10]. O3-type materials have a higher Na content (> 0.85–1) and, therefore, can deliver higher specific capacity. However, these materials face greater migration bottlenecks, as sodium ions must traverse intermediate tetrahedral sites during migration. Additionally, they undergo continuous phase transitions during charge and discharge, driven by the gliding of adjacent TMO_2_ layers to mitigate O–O repulsions [11-13]. To address these challenges and enhance electrochemical performance, various strategies have been developed, including, modulating the crystal structure through various approaches, such as preparing multi-transition metal systems [14-16], creating multiphasic systems [17-19], and surface modification [20, 21].

The representative O3-type layered oxide cathode, α-NaFeO_2_, found in the Earth's crust, was first explored as a potential cathode material for SIBs by Komaba et al. [22] The material exhibited a specific capacity of 80 mAh g^−1^ and a nominal voltage of ~3.3 V within the 2.5–3.5 V range. Subsequent studies on α-NaFeO_2_ revealed that increasing the upper cut-off voltage resulted in high polarization and severe capacity degradation. This degradation is mainly attributed to the migration of Fe from the FeO_6_ layer to the NaO_6_ layer, hindering the reinsertion of Na into the cathode [23, 24]. This phenomenon was also confirmed by Mossbauer studies and DFT calculations on the α-NaFeO_2_ cathode [25]. Despite several strategies to improve the performance of α-NaFeO_2_, the cyclability of this material remains unsatisfactory [26-29].

A monoclinic α-NaMnO_2_ cathode material delivers a reversible capacity of 185 mAh g^−1^ at 0.1C within the 2.0–3.8 V range. The galvanostatic charge-discharge (GCD) curves exhibit multiple plateaus, indicating various phase transformations during cycling, with the material retaining only 70% capacity after 20 cycles [30]. This capacity loss is mainly attributed to the Jahn–Teller distortion of Mn^3+^, often accompanied by multiple transformations in the material [31]. Various strategies have been reported to regulate the Jahn–Teller effect, but improving the cyclability of the cathode remains crucial [32, 33].

Even though layered oxides based on Mn and Fe have high potential for commercial battery applications, their practical application is limited by insufficient capacity and cyclability. Single-transition metal-based layered oxides often undergo several phase transitions during Na-ion intercalation and de-intercalation, resulting in degraded cycle life [11, 34]. Binary transition metal layered oxides based on Fe and Mn are promising candidates, offering excellent electrochemical performance and structural flexibility. P2-Na_2/3_Fe_1/2_Mn_1/2_O_2_ delivers a discharge capacity of 190 mAh g^−1^ in the 1.5–4.3 V range. However, it undergoes a phase transition to OP4 above 4.2 V due to the TM layer sliding, which affects the cycling stability of the material. While Mn^3+^/Mn^4+^ and Fe^3+^/Fe^4+^ redox couples contribute to high capacity, challenges such as air sensitivity and phase instability remain [35-37]. Cathode materials containing Ni have shown great potential for commercial applications based on the Ni^2+/4+^ redox couple, which leads to high capacity [38, 39]. A typical O3-type NaNi_0.5_Mn_0.5_O_2_ has been widely studied, delivering a reversible capacity of 125 mAh g^−1^ within the 2.2–3.8 V range [40]. Although this binary transition metal oxide shows better electrochemical properties, certain shortcomings, such as multiple steps in the GCD profile causing poor cyclability, need to be addressed [41].

Ternary transition metal systems are known to form a solid solution zone through the appropriate addition of elements, which can alleviate the aforementioned issues and improve performance. Layered oxides based on Mn, Fe, and Ni such as NaFe_1/3_Ni_1/3_Mn_1/3_O_2_ are the most widely studied material in the Na(Mn-Fe-Ni)O_2_ family. It shows a capacity of ~130 mAh g^−1^ and decent cyclic performance [42, 43]. Moreover, biphasic Na (Mn-Fe-Ni)O_2_ systems with P2/O3, P2/P3, and ternary phase combinations promise high-voltage stability and prolonged cycle life [44, 45].

**FIGURE 1 ∣:**
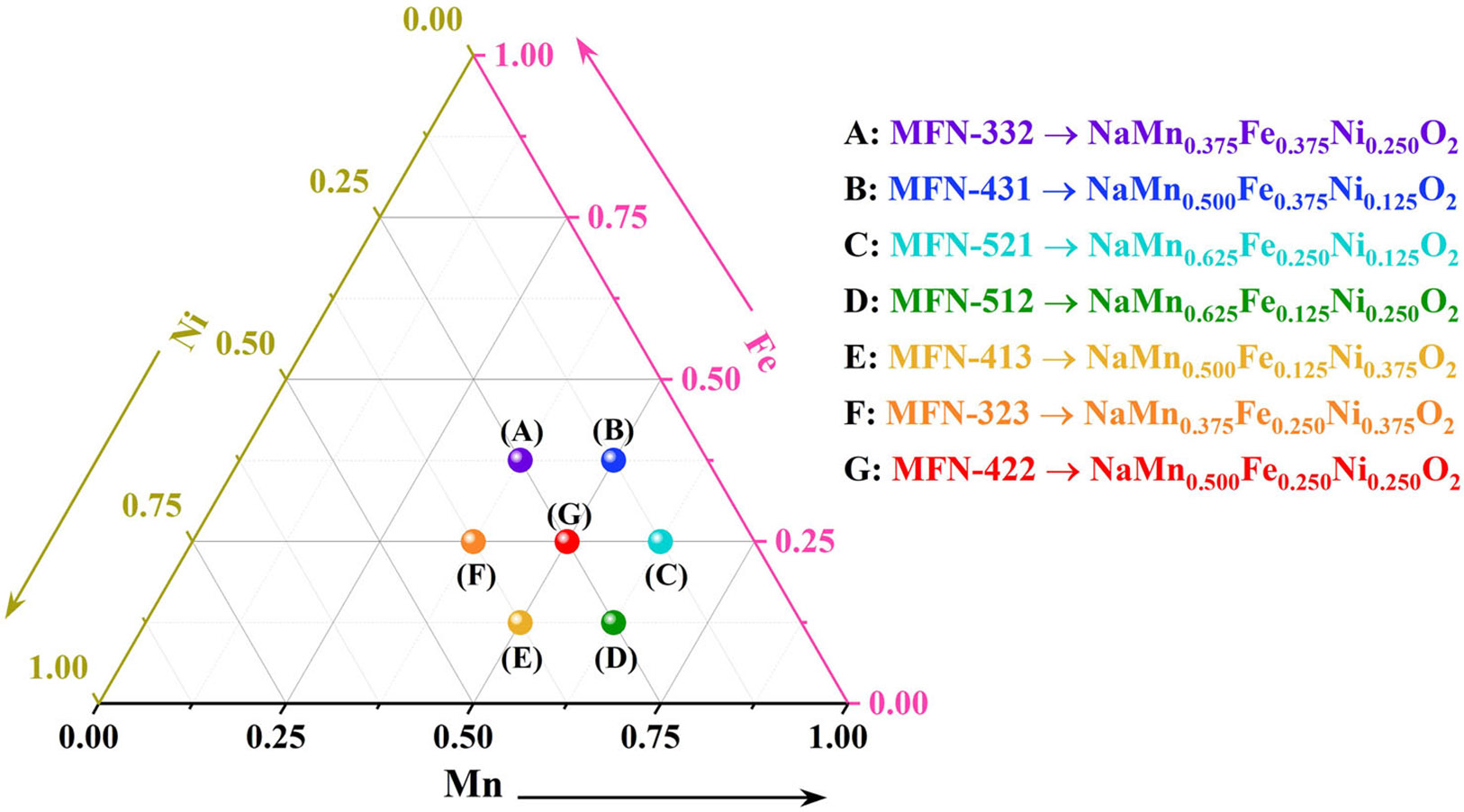
Compositions in the Na(Mn-Fe-Ni)O_2_ pseudo-ternary system studied in this manuscript and their abbreviations.

Many studies have explored the role of transition metal variations and redox inactive dopants in tuning, the charge storage mechanisms, and cycling stability of Fe/Ni/Mn, Fe/Mn, and Mn/Ni-based systems. While prior studies often focus on specific compositions, this work systematically varies Mn, Fe, and Ni ratios to examine their effects on phase formation, structural evolution, and electrochemical behavior [46-61]. Addressing this gap, it explores seven compositions from a pseudo-ternary diagram (Figure 1) based on the formula Na(Mn-Fe-Ni)O_2_, with Mn, Fe, and Ni plotted on the axes. By varying these ratios across a wider compositional range, the study establishes detailed composition–structure–property relationships, providing deeper insights into phase formation, structural evolution, and electrochemical performance. This broader approach advances the understanding of how individual transition metals influence phase stability and electrochemical behavior, distinguishing this work from previous studies.

Although the Ni-rich region offers high specific capacity, it necessitates a high-pressure synthesis procedure to ensure proper Ni diffusion and prevent Ni-rich impurities. Consequently, this region of the phase diagram is excluded from the current study. To minimize the influence of sodium content on phases and resulting properties, all compositions maintain a fixed Na content of 1. Further, to standardize the calcination process, known to affect phase formation (P3 at lower temperatures, P2 and O3 at higher temperatures) [62-65], all samples are calcined only at 900°C. This approach ensures that temperature variations do not introduce additional variables into the study.

X-ray diffraction (XRD) results reveal that although the materials exhibit a preference for the O3 phase due to the Na content being 1, materials with higher Mn content tend to form prismatic sites, leading to the creation of P2/O3 and P2/P3 biphasic compounds. This biphasic intergrowth is confirmed by transmission electron microscopy (TEM) analysis. The smooth GCD curves of the samples suggest a solid solution reaction and enhanced cyclability in these materials. The main objective of this work is to identify compositions in the Na(Mn-Fe-Ni)O_2_ system that exhibit a stable structure, high nominal voltage to enhance energy density, and reduced capacity fading with cycling. A systematic investigation into the effects of Mn, Fe, and Ni concentrations on structural and electrochemical properties led to the identification of MFN-512 as an optimal composition, showing potential for commercialization. MFN-512, characterized by a P2/O3 biphasic structure, can retain 93% of its capacity after 100 cycles at 1 C. The material shows a specific capacity of ~92 mAh g^−1^ in a full-cell configuration with hard carbon as the anode. This study provides a roadmap for systematically optimizing low-cost, energy-dense layered oxides based on Mn, Fe, and Ni.

## 2 ∣ Experimental Section

All pseudo-ternary Na(Mn-Fe-Ni)O_2_ diagram compositions were synthesized using a sol-gel method. Precursors including Na_2_CO_3_ (SRL, 99.9%), C_4_H_6_MnO_4_·4H_2_O (Sigma-Aldrich, > 99%), Fe(NO_3_)_3_·9H_2_O (Rankem. 98%), and C_4_H_6_NiO_4_·4H_2_O (Sigma-Aldrich, 98%) were measured in stoichiometric amounts and mixed in deionized water. This mixture was stirred for 8 h before adding citric acid (C_6_H_8_O_7_) as a chelating agent and ethylene glycol (C_2_H_6_O_2_) as a gelling agent. The resulting solution was continuously stirred to form a homogeneous mixture and then heated to form a gel. The gel was dried and, subsequently, heated at 550°C, followed by calcination at 900°C in a muffle furnace under ambient air. After calcination, the products were allowed to cool naturally and then transferred to an argon-filled glove box to prevent moisture exposure from the air.

Phase identification was performed by XRD using an Empyrean instrument from Malvern Panalytical with Cu-Kα radiation. The XRD data were collected over a 2θ range of 10°–70° with a step size of 0.01° and analyzed using Rietveld refinement with Topas academic software (version 6) [66]. Morphological studies of the samples were conducted using field emission scanning electron microscopy (FESEM) with a JEOL-7610 model. Energy dispersive X-ray spectroscopy (EDS) was employed to investigate the elemental distribution within the samples. TEM studies were performed using a JEOL-2100 TEM. For these studies, powder samples were drop-cast on the carbon-coated Cu grids. X-ray photoelectron spectroscopy (XPS) using a Thermo Fisher Scientific instrument with a 1486.6 eV (Al Kα) X-ray source was utilized to determine the oxidation states of elements in the cathode materials.

The positive electrodes were prepared by coating a slurry composed of 75% active material, 10% Ketjen black, and 15% polyvinylidene fluoride (PVDF) binder, all by weight, in N-methyl-2-pyrrolidone (NMP) solvent. This slurry was coated to an aluminum (Al) current collector, dried under vacuum, and punched into 16 mm discs to achieve an active material loading between 3 and 4 mg cm^−2^. CR2032 coin cells were assembled in an argon-filled glove box, using the prepared electrodes and sodium metal as the negative electrode. To fabricate the full cell, hard carbon was employed as the anode material, coated onto the aluminum foil with a mixture containing 80 wt% hard carbon, 10 wt% Ketjen black, and 10 wt% PVDF as the binder. Before its incorporation as the negative electrode in the MFN-512—hard carbon full cell, the hard carbon electrode was presodiated through electrochemical sodiation and de-sodiation in a sodium metal half-cell setup. The electrolyte used was 1 M NaClO_4_ in a 1:1 mixture of ethylene carbonate (EC) and propylene carbonate (PC), with Whatman GF/D as the separator.

GCD experiments were conducted at various current densities using a Neware battery tester (Model CT-4008T). The same equipment was used to perform the galvanostatic intermittent titration technique (GITT) and calculate the diffusion coefficient of Na-ions in the cathode material. Electrochemical impedance spectroscopy was carried out on the coin cells using an NF Corp. LCR meter (Model ZM 2376) by applying a perturbation voltage of 10 mV over a frequency range of 1 MHz to 100 mHz. The phase transformations that occurred during electrochemical cycling were investigated by operando studies carried out using extreme conditions-angle dispersive/energy dispersive synchrotron XRD (BL11) at Indus-2 beamline (RRCAT) with a beam wavelength of 0.7388 Å and beam energy of 2.5 GeV. The coin cells for the operando studies were prepared by drilling 5 mm holes in the CR2032 coin-cell casing to allow the beam to pass through the cell. A Kapton tape was used to cover the drilled holes in coin cell casings to prevent environmental exposure.

## 3 ∣ Results and Discussion

Phase identification and purity of the samples were confirmed using XRD. Supporting Information: Figure S1 presents the XRD patterns of the MFN samples. The analysis of the XRD data indicates that all peaks correspond to the Bragg positions of the O3, P3, and P2 phases, and no impurity peaks were observed. The patterns demonstrate a clear preference for the O3 phase across all samples, which is expected due to full Na occupancy. As the Mn content increases (in MFN-512 and MFN-521), the compounds exhibit biphasic behavior, incorporating P2 and P3 phases. Given that Mn has lower electronegativity (1.55 on the Pauling scale) than Fe (1.83) and Ni (1.91), the repulsion between oxygen ions increases, favoring the formation of prismatic sites over octahedral sites. Specifically, MFN-512 exhibits (002), (102), and (103) peaks, which can be indexed to the P2 phase, in addition to peaks of the O3 phase, making MFN-512 a biphasic compound with P2/O3 phases. Similarly, MFN-521 is identified as a biphasic compound comprising P2/P3 phases (Supporting Information: Figure S1). The low-intensity peaks at ~20° (marked by * in Supporting Information: Figure S1) are attributed to transition metal (TM) ordering. The TM ordering in layered oxides arises from dissimilar oxidation states and ionic radii of the constituent transition metals [67].

**FIGURE 2 ∣:**
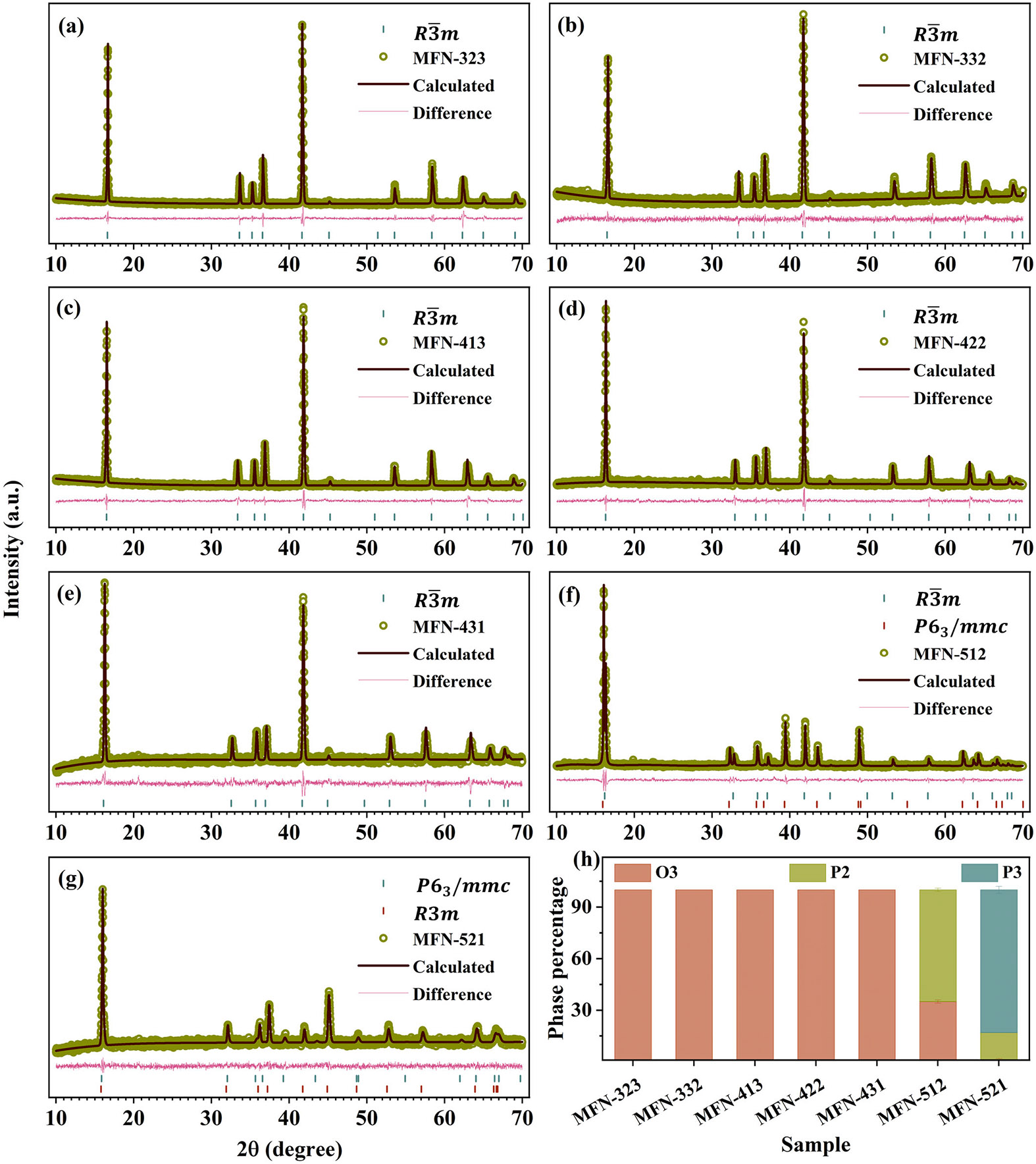
(a–g) Rietveld refined patterns of MFN samples. (h) Phase percentage of the various phases in MFN samples.

The Rietveld refinement of the XRD data shows that the P2, P3, and O3 phases correspond to the P6_3_/*mmc*, *R*3*m*, and $R\bar{3}m$ space groups, respectively (Figure 2). Phase quantification reveals that MFN-512 consists of 65% P2 and 35% O3 phases, while MFN-521 has a P2:P3 ratio of 17:83. The lattice parameters obtained from the refinement are presented in Table 1. With an increase in Ni content, there is an overall decrease in the c parameter. This is contrary to the expected increase in the c parameter with increasing Ni, as the ionic radius of Ni^2+^ in six-coordination (0.69 Å) is larger than that of Mn^3+^ (0.58 Å), Mn^4+^ (0.53 Å), and Fe^3+^ (0.55 Å). For samples on constant Ni lines, the c parameter increases with an increasing Mn/Fe ratio. This suggests that the effects of the higher electronegativity of transition metal ions on the lattice parameters are more significant than the effects of larger ionic radii in these samples. With increasing Mn content, there is an overall increase in the c parameter, and for the samples on constant Mn lines, the c parameter increases with an increasing Fe/Ni ratio. These observations suggest a complex interplay between the transition metals in layered oxides, where increased Ni content contracts the lattice, while, an increase in Mn and Fe content has a relatively expanding effect on the c parameter, depending on their ratios. The refinement parameters of all the samples are presented in Supporting Information: Tables S1-S7.

Further, the transition metal layer spacing ($S_{\text{TMO}2}$) and the sodium layer spacing ($S_{\text{NaO}2}$) in the O3 phase can be calculated using the following Equations (1) and (2), respectively:

(1) $$S_{{\text{TMO}}_{2}}=\left(\frac{1}{3}-z_{\text{ox}}\right)2c_{\text{hex}}$$

(2) $$S_{{\text{NaO}}_{2}}=\frac{c_{\text{hex}}}{3}-S_{{\text{TMO}}_{2}}$$

Here, $z_{\text{ox}}$ and $c_{\text{hex}}$ denote the fractional coordination z of the oxygen ions and the c parameter, respectively. The results are summarized in Supporting Information: Table S8. Samples with higher Mn content and a Ni/Fe ratio > 1 have higher S_NaO2_, indicating a larger sodium layer spacing compared to others. It is important to note that the rate performance of layered oxides largely depends on the facile conduction of Na^+^ in the sodium layer. A larger Na layer spacing would facilitate the migration of Na-ions through the material, allowing it to perform better at higher C rates. Accordingly, it is expected that MFN-431, MFN-512, and MFN-422 will exhibit better rate performance compared to the other materials considered in this study.

**FIGURE 3 ∣:**
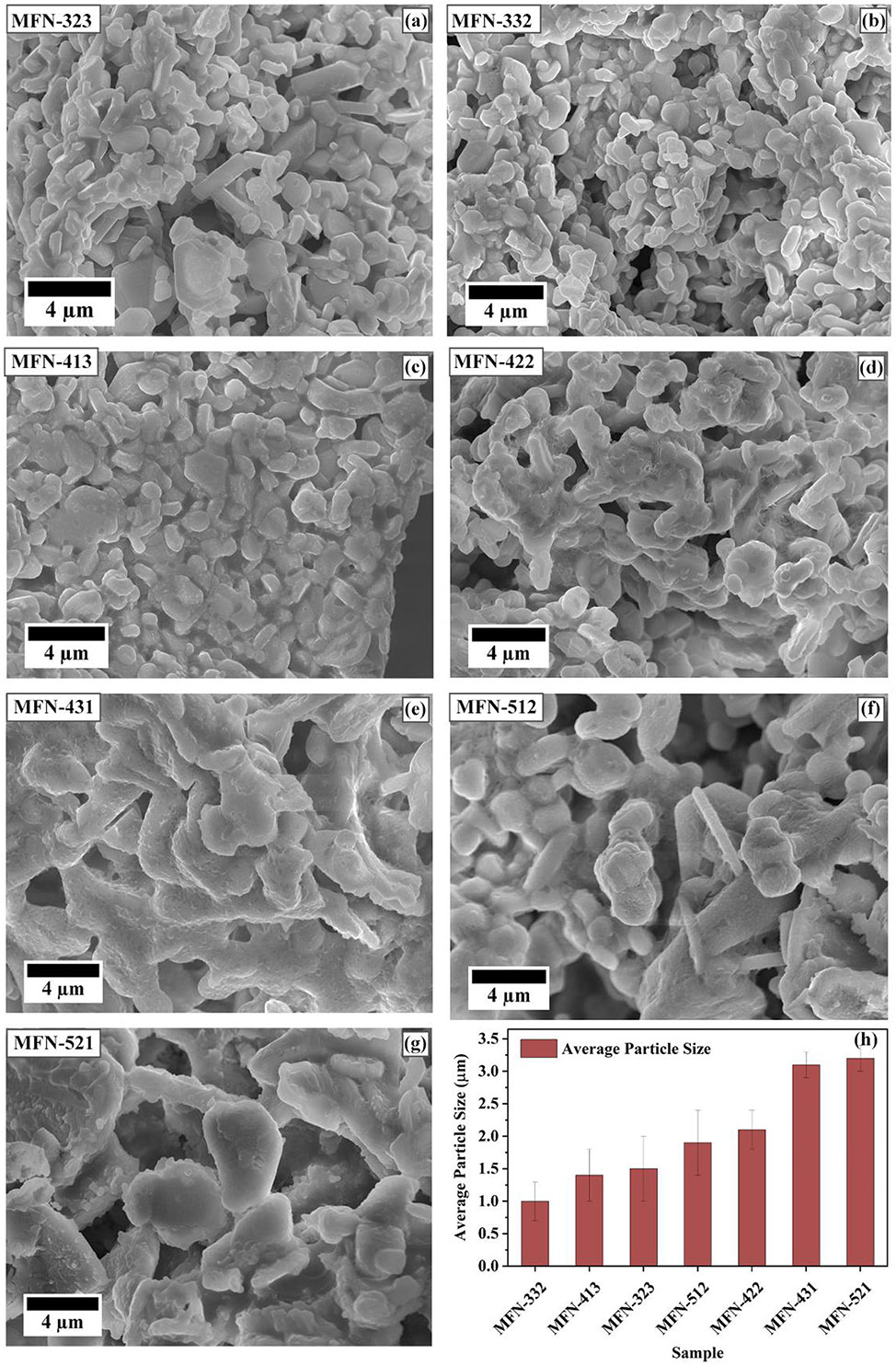
(a–g) SEM micrographs of MFN samples. (h) Variation in the average particle sizes of the materials.

A study of the morphological and microstructural characteristics of MFN samples was conducted using FESEM, and the SEM micrographs are presented in Figure 3. These images reveal that the particles are disc-shaped, which can be attributed to the highly anisotropic crystal structure of layered oxides. A comparison of the average particle size indicates that, generally, increasing the fractions of Fe and Mn results in larger, less agglomerated particles. Conversely, a higher Ni content tends to produce smaller, more agglomerated particles. Smaller particles in cathode materials are generally less prone to cracking, leading to better cyclability. Additionally, a lower degree of agglomeration enhances performance at higher discharge rates. Therefore, an optimal balance between particle size and degree of agglomeration must be achieved for improved performance. The EDS mappings of MFN-413 and MFN-512 in Supporting Information: Figure S2 confirm that all elements are homogeneously distributed throughout the samples.

**FIGURE 4 ∣:**
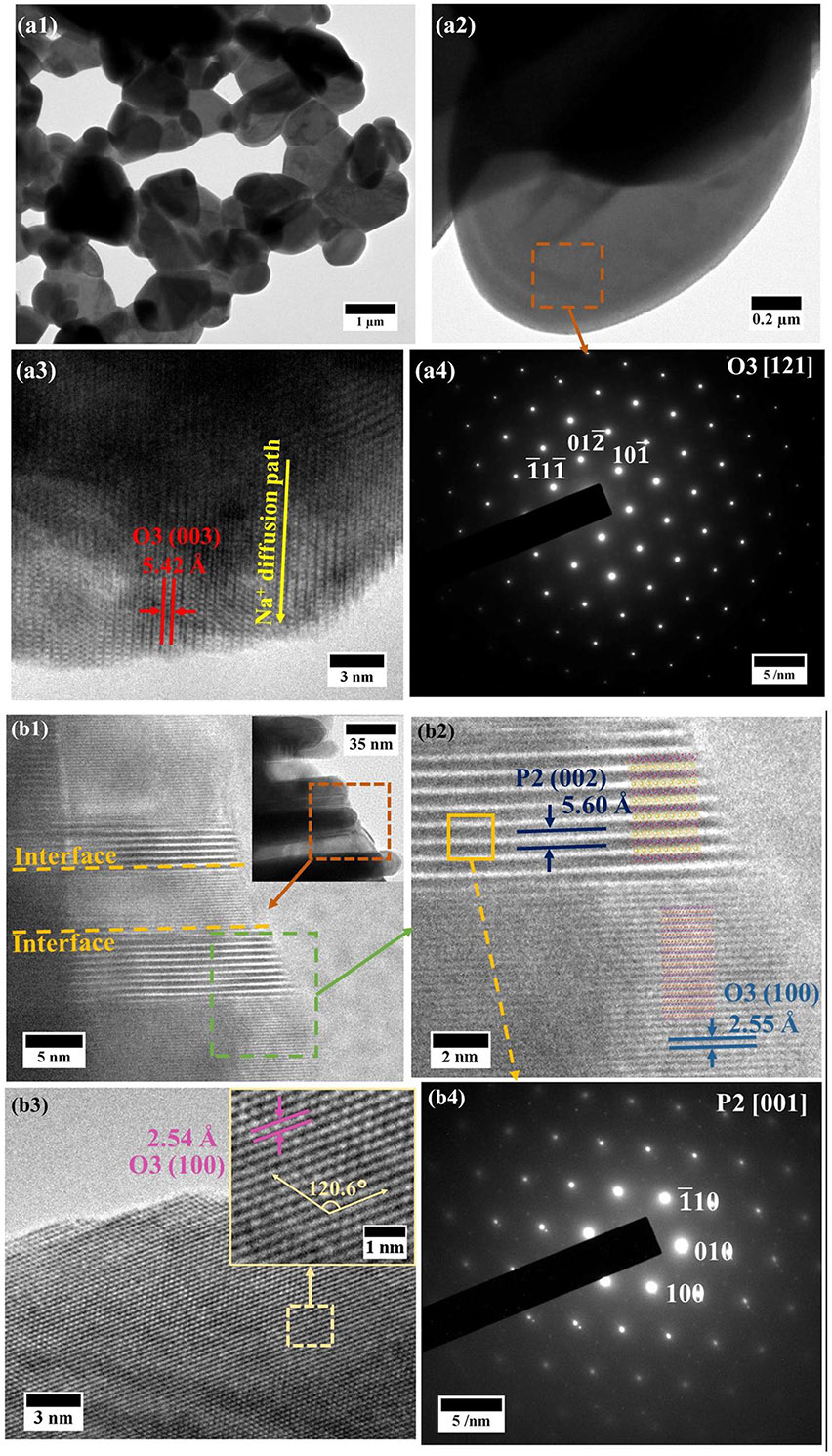
(a1), (a2) Bright-field HRTEM images of MFN-422, (a3) atomic resolution image of MFN-422, and (a4) SAED pattern of MFN-422 captured along the [121] zone-axis. (b1), (b2) HRTEM images of MFN-512 (inset (b1) shows intergrowth of P2 and O3 phases, (b3) atomic resolution image of MFN-512 (inset is a magnified image showing the hexagonal arrangement of atoms), and (b4) SAED pattern of MFN-512 captured along the [001] zone-axis.

The local structure of the MFN samples was further examined using TEM. The bright field images, high resolution (HRTEM) images, and selected area electron diffraction (SAED) patterns of MFN-422 and MFN-512 samples are shown in Figure 4. The lattice fringes in Figure 4(a3) for the sample MFN-422 display an interplanar spacing of ~5.42 Å, attributed to the (003) plane of the O3 phase. Figure 4(a4) shows the SAED pattern captured along the [$12\bar{1}$] zone-axis shows the diffraction spots that can be indexed to the ($10\bar{1}$), ($01\bar{2}$), and ($\bar{1}1\bar{1}$) planes of O3 NaMn_0.5_Fe_0.25_Ni_0.25_O_2_, aligning well with the atomic arrangement of the $R\bar{3}m$ space group.

The low-magnification TEM image of MFN-512 in Figure 4(b1) inset reveals the presence of two intergrown distinct phases. A high-resolution image shows the lattice fringes with different interplanar spacings. One set of fringes shows an interplanar spacing of 5.60 Å, corresponding to the (002) plane of the P2 phase, while another set exhibits an interplanar spacing of ~2.55 Å, corresponding to the (100) plane of the O3 phase. The crystal structures of the P2 and O3 phases, generated using VESTA, are overlaid on the magnified image to better visualize the arrangement of NaO_2_ and TMO_2_ layers in the different phases. An atomic resolution image in Figure 4(b3) displays an interlayer spacing of ~2.54 Å, corresponding to the (100) plane of the O3 phase. The SAED pattern in Figure 4(b4) shows hexagonally arranged diffraction spots, indexed to the (010), ($\bar{1}10$), and (100) planes of the P2 phase of the NaMn_0.625_Fe_0.125_Ni_0.25_O_2_. The faint superlattice spots in the SAED pattern (Figure 4b4) correspond to the TM honeycomb ordering, as reported in previous studies [68].

**FIGURE 5 ∣:**
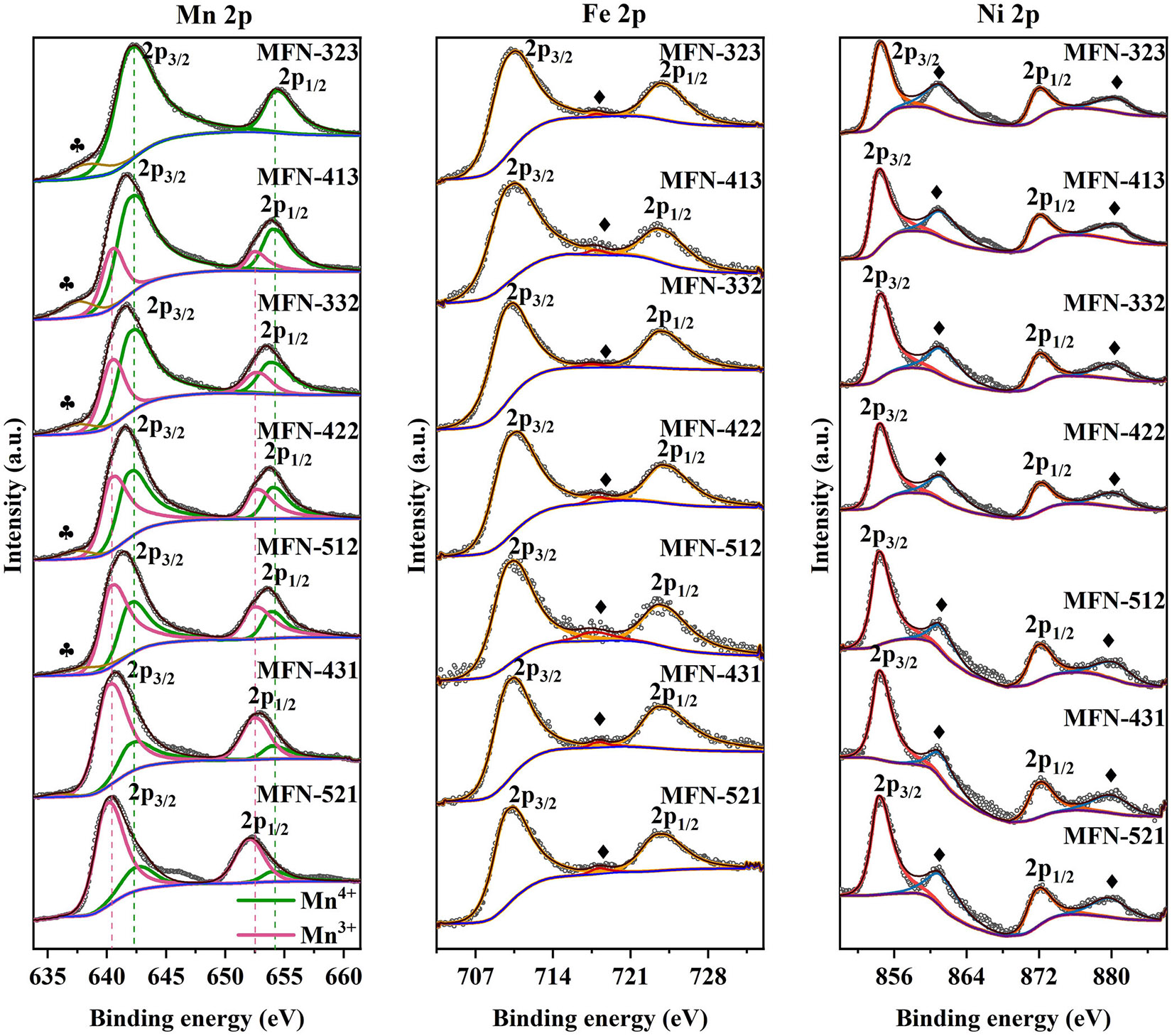
Mn 2p spectra, Fe 2p spectra, and Ni 2p spectra of the MFN samples. ♣ represents the Auger peaks in the Mn 2p spectra, and ♦ represents the shake-up satellite peaks in the Fe 2p and Ni 2p spectra.

The oxidation states of Mn, Fe, and Ni in the MFN samples were verified using XPS (Figure 5). The Mn 2p spectrum of MFN-323 displays two peaks at approximately 642.6 and 653.7 eV, corresponding to Mn 2p_3/2_ and Mn 2p_1/2_, respectively. These binding energy values indicate the presence of tetravalent Mn, confirming Mn^4+^ in MFN-323. Moving towards the right side of the triangle in Figure 5, the Mn spectra of the samples can be deconvoluted into four peaks: two for Mn^3+^ (Mn 2p_3/2_ at ~641.2 eV and Mn 2p_1/2_ at ~651.9 eV) and two for Mn^4+^ (Mn 2p_3/2_ at ~642.6 and Mn 2p_1/2_ at ~653.7 eV) [56, 69, 70]. Additionally, the ratio of Mn^3+^ to Mn^4+^ increases towards the right of the triangle. The broad peak at ~636 eV can be attributed to the Auger peak in the Mn 2p spectrum (marked by ♣ in the Mn 2p spectra in Figure 5) [71]. The Fe 2p spectrum shows two coupled peaks at 710.7 and 724.2 eV, suggesting the presence of Fe^3+^ ions in all MFN samples [56, 69, 70]. The Ni spectrum shows peaks belonging to Ni 2p_3/2_ and Ni 2p_1/2_ at 854.4 and 872.1 eV, respectively, along with their shake-up satellites (marked by ♦ in the Ni 2p spectra in Figure 5), confirming the presence of Ni in the 2+ oxidation state in all MFN samples [56, 69, 70].

**FIGURE 6 ∣:**
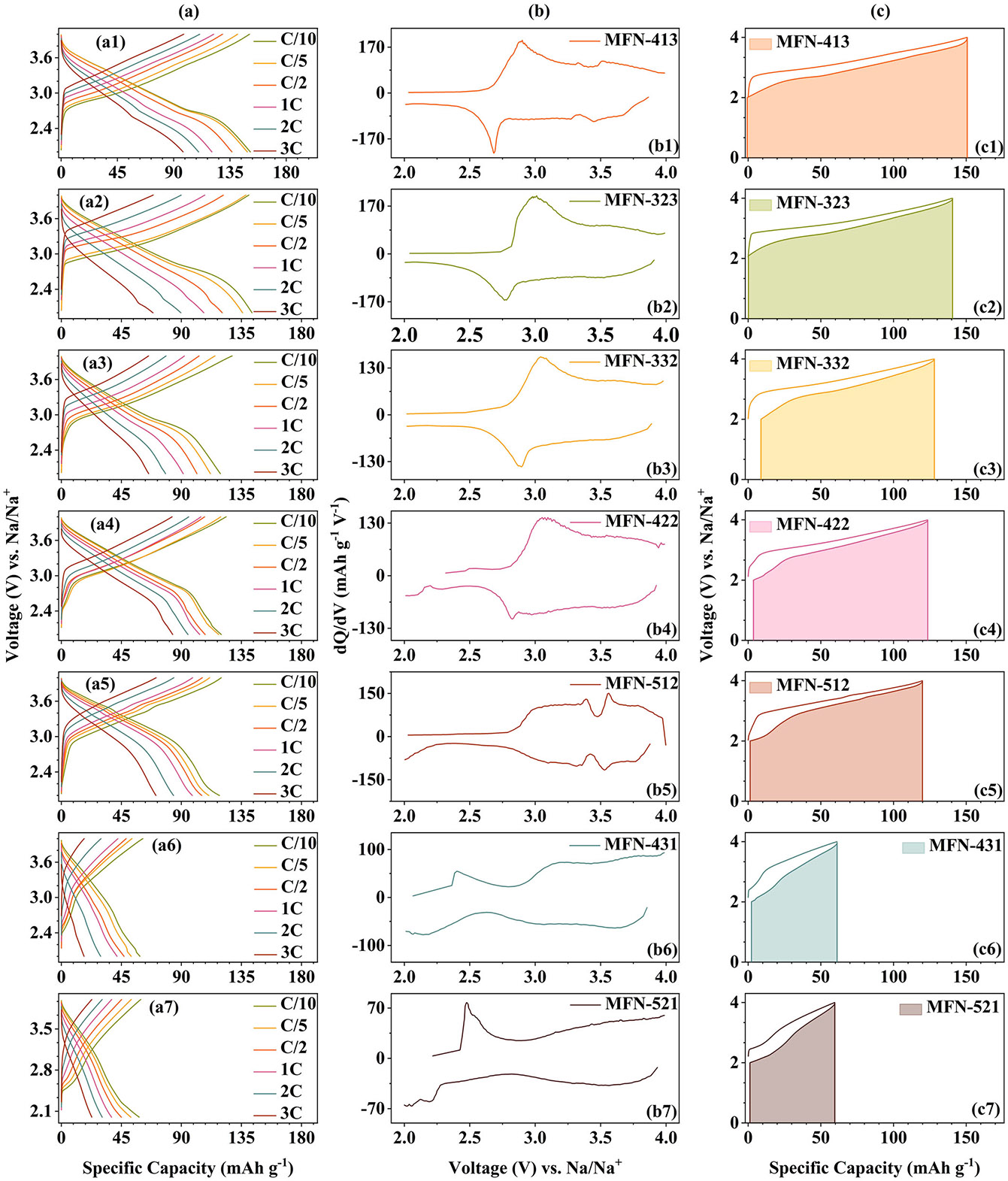
(a) Galvanostatic charge-discharge curves at different C-rates, (b) *dQ/dV* versus V profiles, and (c) Energy efficiency curves of MFN samples.

The effects of the phases and structural parameters on the electrochemical properties of MFN samples were studied, and the results in the subsequent discussion are summarized in Table 2. The GCD profiles at different C-rates are shown in Figure 6(a), and the corresponding *dQ/dV* versus V curves at 0.1C are presented in Figure 6(b). The first discharge specific capacities of the MFN cathodes are tabulated in Table 2. As the Ni content decreases, the specific capacity at 0.1C decreases. Furthermore, with increasing Fe content, the GCD curves become more sloping, with the plateaus above 3.2 V merging together. In the *dQ/dV* versus V curves for MFN-413 and MFN-512, in addition to the broad peaks at ~3.1/2.9 V, peaks corresponding to Na-ion/vacancy ordering rearrangements that occur during cycling also appear at ~3.6/3.5 V, whereas the Na-ion/vacancy ordering peaks are absent in other compositions. This indicates that an increase in Fe content results in a solid solution reaction and the absence of Na^+^/vacancy ordering upon sodiation/desodiation [49, 58]. It is generally believed that Na-ion/vacancy ordering negatively impacts the cathode's structure and Na-ion diffusion kinetics. However, a recent study by Wang et al. established that a dual-ordered structure with both Na-ion vacancy and TM ordering enhances the cathode's working voltage and structural stability [68]. The average voltage values of the MFN samples are given in Table 2. These values are in accordance with the above discussion, where MFN-512 has a higher average voltage compared to others. The specific discharge capacities of MFN-413, MFN-323, MFN-332, MFN-422, MFN-512, MFN-431, and MFN-521 at 0.1C in second cycle are approximately 149, 142, 120, 119, 120, 58, and 57 mAh g^−1^, respectively.

MFN-521 and MFN-431, with Ni/Fe ratio < 1, exhibit relatively higher polarization, which further increases at higher C-rates. High polarization implies slower Na-ion kinetics, which can negatively impact the long-term cyclability of the material. This higher polarization could be due to several factors, including larger particle sizes and a Ni/Fe ratio being < 1 [72]. Several cathodes containing Ni and Fe have shown suppressed polarization with increasing Ni/Fe ratio, a trend that is also observed in this study [8]. The energy efficiency of MFN cathodes at 0.1C is shown in Figure 6(c), with values given in Table 2. The shaded area under the curve represents the amount of energy recovered during discharge compared to the total energy input during charging. MFN-512 sample also shows a remarkable energy efficiency of ~91.3%.

**FIGURE 7 ∣:**
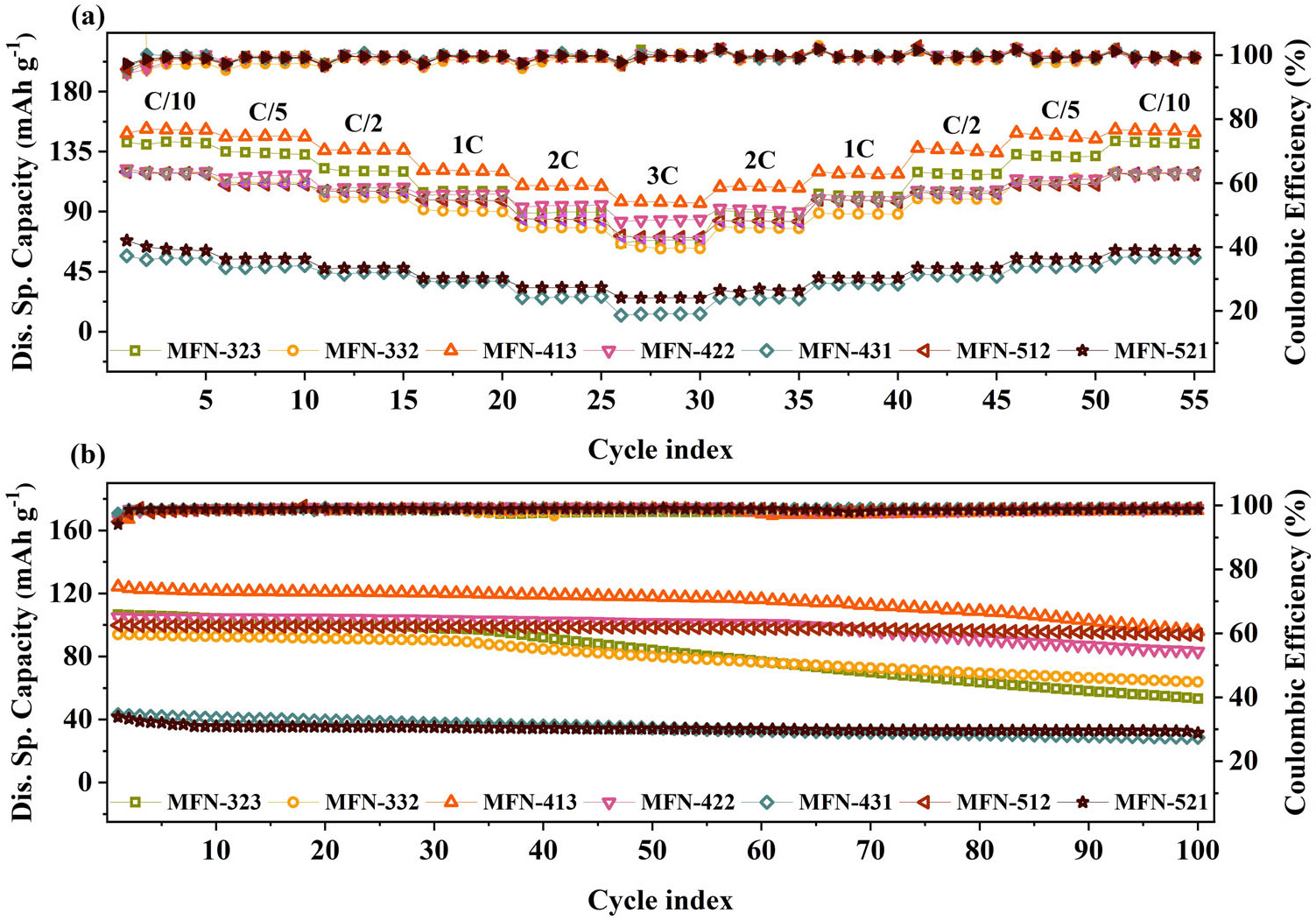
(a) Rate performance and (b) cyclic stability of MFN samples.

Figure 7a displays the rate performance of MFN samples at various C-rates in the 0.1 C to 3 C range. All samples nearly recover their original discharge capacities when charged from higher to lower C-rates, indicating no irreversible loss of capacity with C-rate. The percentage capacity retention at 1C and 3C compared to 0.1C is summarized in Table 2. There are substantial variations in rate performance due to the different local environments created by varying transition metal ratios in the samples. Although materials with the O3 phase are reported to have poor rate performance due to slower Na-ion kinetics through the tetrahedral sites, MFN-422 and MFN-413 with pure O3 phase show excellent rate performance. MFN-422 (NaMn_0.500_Fe_0.250_Ni_0.250_O_2_) demonstrated the best rate performance, exhibiting 85.7% capacity at 1C and 69.7% at 3C, compared to 0.1C. Also, MFN-512, a biphasic compound with both P2 and O3 phases, delivered an excellent capacity retention of ~83% at 1C of the capacity at 0.1C. The exceptional rate performance in MFN-422, MFN-413, and MFN-512 is attributed to the larger Na-layer spacing in these materials, which facilitates efficient Na-ion conduction. Apart from the Na^+^ diffusion inside the crystal structure, the rate performance of a cathode material is also determined by the particle size and degree of agglomeration. In this regard, MFN-413, MFN-422, and MFN-521, with smaller average particle sizes, perform better than MFN-431 and MFN-521. The rate performance of MFN-512 is further complemented by its biphasic nature, as P2 phases are known to facilitate faster Na-ion diffusion.

The cyclability of MFN samples at 1C is shown in Figure 7(b) and listed in Table 2. MFN-512, MFN-422, and MFN-413 retain nearly ≥ 80% of their initial capacity after 100 cycles. Cyclability data shows that when Mn content is high, and the Ni/Fe ratio is > 1, such materials have better cyclability. Higher capacity retention in these materials can be attributed to lesser agglomeration and optimal particle sizes, which maintain structural integrity.

The exceptional cyclability of MFN-512 is also attributed to the widely reported interlocking effect in biphasic oxides, which stabilizes the structure during cycling [44, 45, 73, 74]. TEM analysis confirms the presence of intergrown P2 and O3 phases in MFN-512. Such biphasic structures are known to strengthen structural integrity by preventing layers from gliding over one another and, thus, improving the cyclic stability of cathodes. The properties of various cathode materials in the Na(Mn-Fe-Ni)O_2_ system are compared in Supporting Information: Table S9. This table highlights the competitive performance of MFN-512 and demonstrates its potential for practical applications. Although MFN-521 also shows decent capacity retention, its low specific capacity limits its practical applications.

Overall, a decreasing Ni content results in lower specific capacity, while an increasing Fe content leads to more sloped GCD curves and suppresses Na-ion/vacancy ordering. Smaller particle sizes in MFN-413, MFN-422, and MFN-521 improve rate performance. Additionally, the biphasic structure of MFN-512 enhances cyclability, providing superior cyclability. These findings underscore the pivotal role of phase composition and transition metal ratios in optimizing the electrochemical performance of sodium-ion battery cathodes, leading to enhanced rate capability, higher specific capacity, and improved long-term stability.

**FIGURE 8 ∣:**
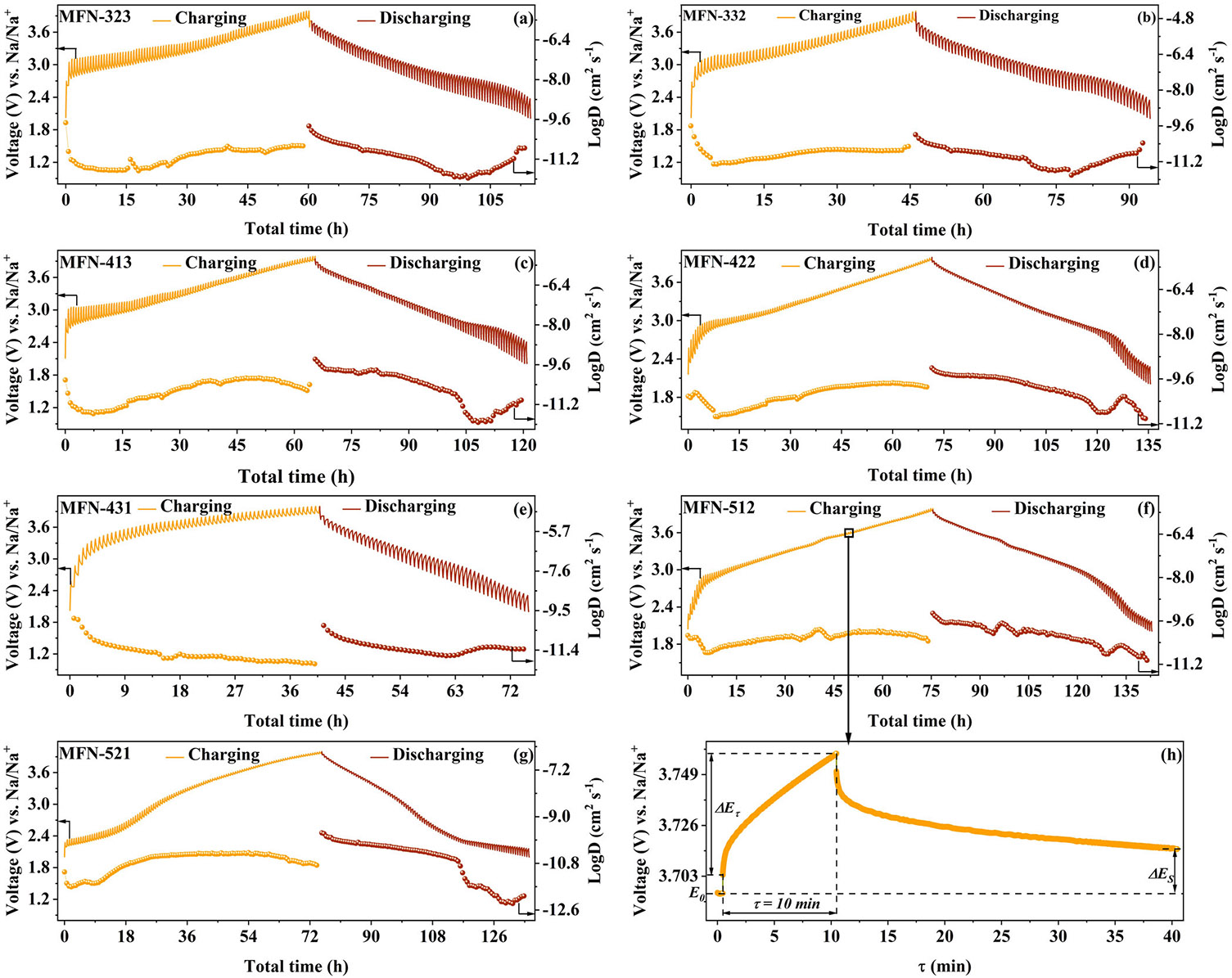
(a–g) GITT profiles of MFN samples at 0.1C along with the variations in diffusion coefficients during charge-discharge. (h) A single titration unit of the GITT curve of MFN-512 during charging shows the variables considered in Equation (1).

The diffusion coefficient of Na-ions (D_Na+_) in the MFN cathodes was determined using the GITT. The GITT curves and corresponding diffusion coefficient values are presented in Figure 8. Before conducting GITT, the cells underwent a first formation cycle. For the GITT measurements, cells were subjected to a constant current pulse at 0.1C for 10 min, followed by a 30-min relaxation period to allow the cathodes to reach a pseudo-equilibrium state. The diffusion process is assumed to follow Fick's first law of diffusion. The D_Na+_ values are calculated using the following Equation (3) [75]:

(3) $$D_{{\text{Na}}^{+}}=\frac{4}{\pi \tau }{\left(\frac{m_{B}V_{m}}{M_{B}S}\right)}^{2}{\left(\frac{\Delta E_{S}}{\Delta E_{\tau }}\right)}^{2}\;(t\ll L^{2}∕D)$$

where:

- τ is the duration of the constant current pulse,
- $m_{B}$ is the active material loading on the cathode,
- $V_{m}$ is the molar volume of the material,
- MB is the molecular weight of the material,
- S is the surface area of the cathode,
- $\Delta E_{\tau }$ is the voltage change during the current pulse,
- $\Delta E_{S}$ is the voltage change when the material reaches equilibrium.

The calculated D_Na+_ values for all samples are tabulated in Supporting Information: Table S10. Analysis reveals that the diffusion coefficients are higher in the better-performing MFN-512 and MFN-422 samples. Figure 8 shows that Na-ion diffusion slows down at higher and lower voltages when the cathode becomes either deficient or saturated with Na-ions, indicating slower kinetics in these regions [71, 76, 77]. Notably, the biphasic MFN-512 exhibits a higher D_Na+_ value of 4.0 V compared to the other samples. This highlights the advantage of the biphasic material with optimal Mn/Fe/Ni ratios in enhancing electrochemical performance.

**FIGURE 9 ∣:**
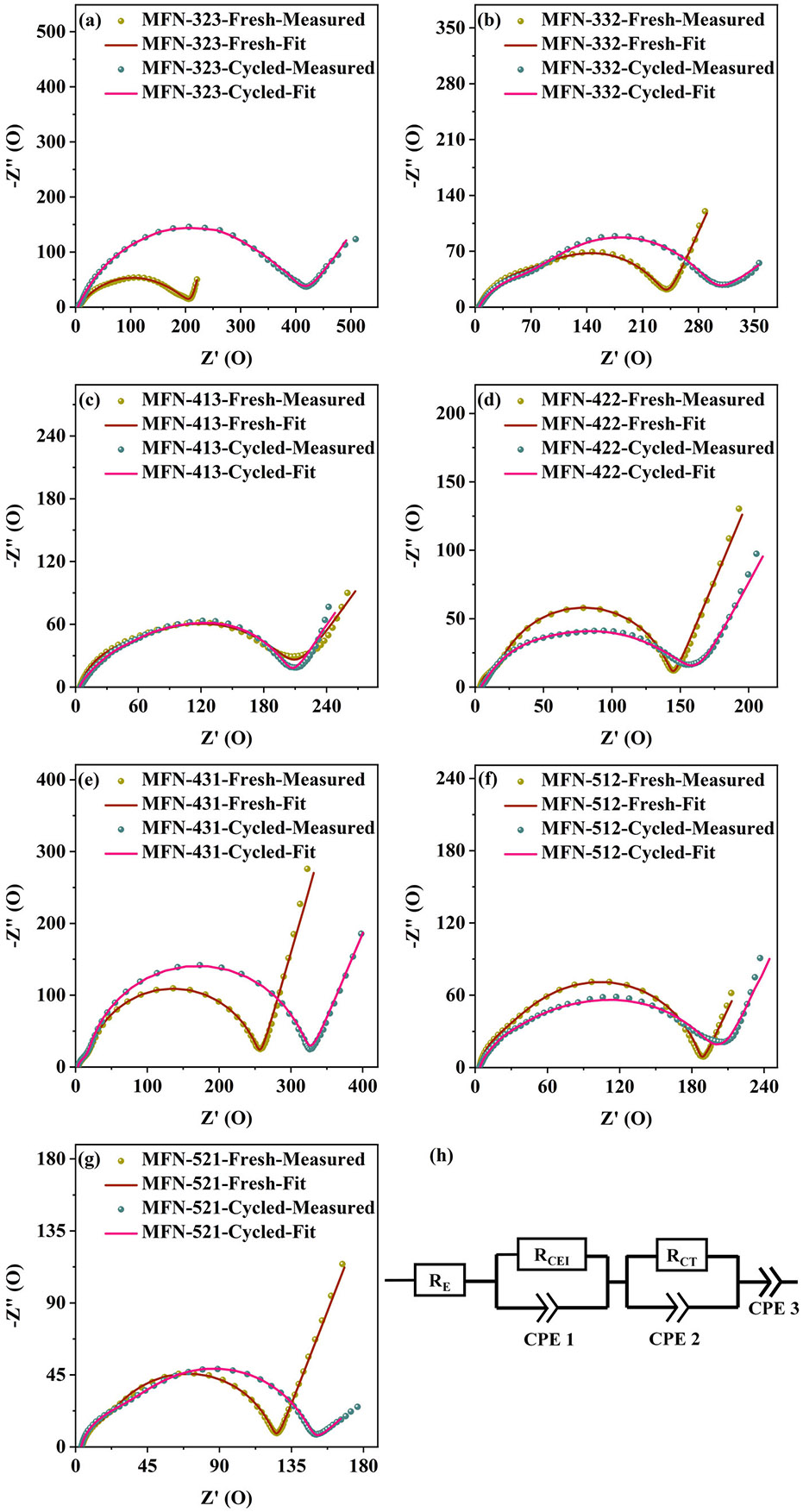
(a–g) Nyquist plots of MFN samples before and after cycling along with their fitted curves. (h) Circuit model for fitting.

The cells with MFN cathodes were analyzed using electrochemical impedance spectroscopy (EIS) both before and after 100 charge/discharge cycles to investigate the superior electrochemical properties of MFN-413, MFN-422, and MFN-512. EIS data, measured over a frequency range from 1 MHz to 100 mHz with a perturbation voltage of 0.01 V, are illustrated in Figure 9(a-g) in the form of Nyquist plots for all samples. The corresponding equivalent circuit model used for fitting is shown in Figure 9(h). The Nyquist plots feature an intercept on the Z′-axis at high frequencies, representing the electrolyte resistance (*R*_E_). Two overlapping semi-circular arcs are observed, corresponding to the resistance of the CEI layers formed on the electrode surfaces (*R*_CEI_) and the charge-transfer resistance (*R*_CT_). The tail observed in the low-frequency region reflects impedance associated with ion diffusion in the cathodes. A close match between the experimental and fitted curves is observed for all samples with associated errors of < 1% in observed values of resistances. The fitted resistance values for the MFN samples are summarized in Supporting Information: Table S11.

The resistance values reveal that all samples have an electrolyte resistance ranging from 3.5 to 4.1 Ω, which remains relatively stable even after cycling. This stability suggests that there are no parasitic reactions between the electrodes and the electrolyte, and the electrolyte's homogeneity is preserved. Samples with smaller particle sizes exhibit higher *R*_CEI_ values due to the increased surface area, which leads to more CEI formation. The CEI resistance shows only a marginal increase after cycling, indicating no significant particle cracking or new surface formation that would enhance CEI growth. The charge transfer resistance for samples with lower cyclability, particularly MFN-323, increases significantly, causing a prominent second semi-circular arc and a ~109% increase in total resistance. In contrast, MFN-413, MFN-422, and MFN-512 samples demonstrate relatively lower charge transfer resistances, which show relatively small changes with cycling, facilitating easier Na-ion diffusion within the material as indicated by the higher D_Na+_ values calculated from GITT. This results in reduced polarization and better cyclability for these samples. Moreover, MFN-512, with its P2/O3 biphasic structure, enables more efficient sodiation and desodiation, exhibiting a minimal change (~6.6%) in total resistance.

**FIGURE 10 ∣:**
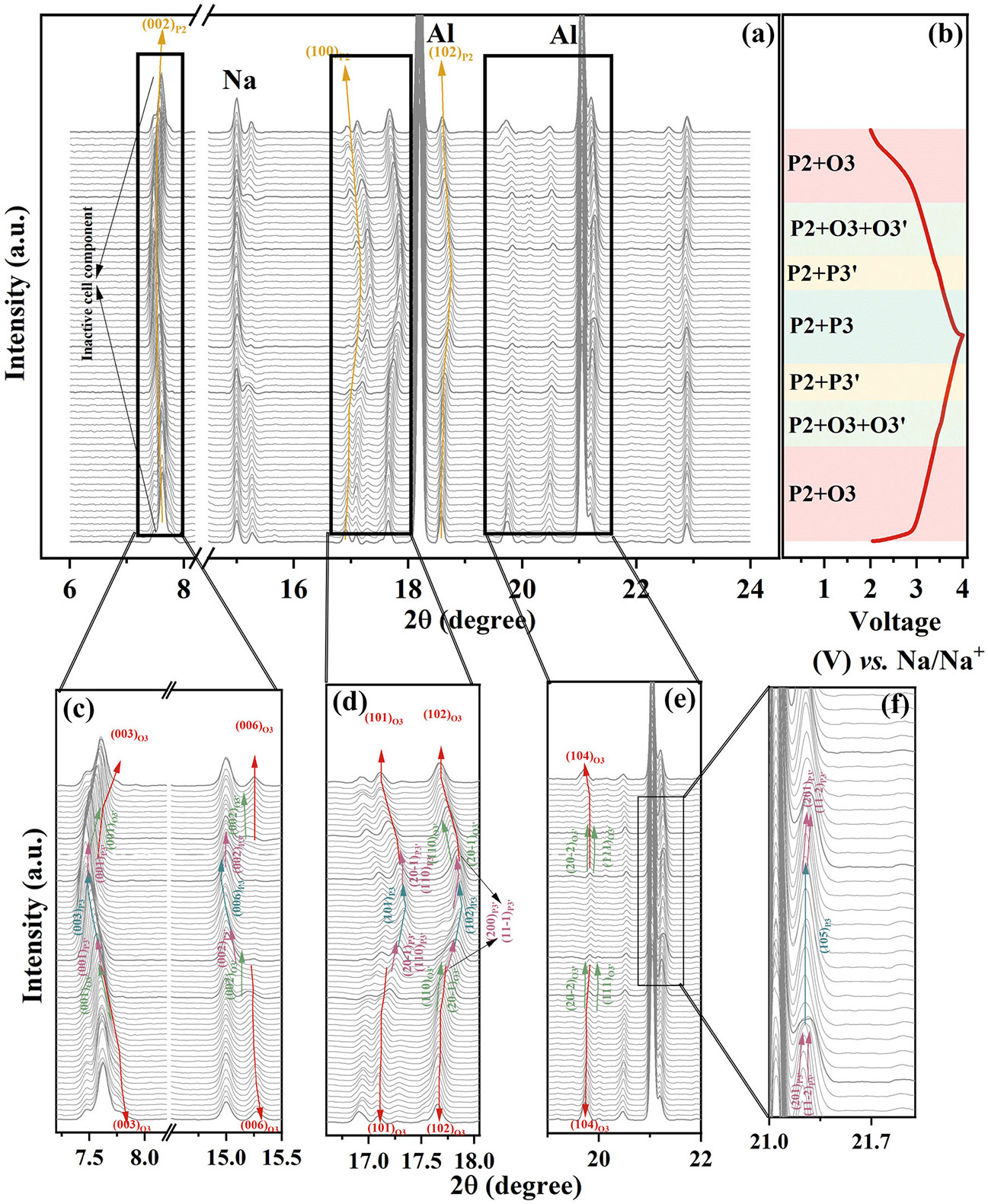
(a) *Operando* synchrotron XRD of MFN-512. (b) Corresponding GCD curve showing various states of charge/discharge. Magnified view of (c) (002)_P2_ & (003)_O3_, (d) (100)_P2_, (101)_O3_ & (012)_O3_, (e) (102)_P2_ & (104)_O3_, and (f) (105)_O3_ peaks of MFN-512 showing various phase transformations in the O3 phase.

Operando synchrotron x-ray diffraction (SXRD) was employed to elucidate the structural evolution of MFN-512 during charging/discharging. Figure 10(a) presents SXRD patterns recorded at a charge/discharge rate of 0.2C in the 2.0–4.0 V range, while the corresponding GCD profile is shown in Figure 10(b). During charging, the (003) and (006) reflections of the O3 phase systematically shift to lower angles, indicative of the expansion of the c parameter driven by the glide of TMO_2_ layers to mitigate electrostatic repulsion (Figure 10c). At ~3.3 V, the O3 phase transitions to the monoclinic O3′ phase, as evidenced by the emergence of (001) and (002) reflections. On further charging, a monoclinic P3′ phase emerges at ~3.6 V, confirmed by the (201) and ($11\bar{2}$ reflections in Figure 10(f). This phase subsequently transforms into the hexagonal P3 phase, as indicated by a marked decrease in the (104) peak intensity and the increased intensity of the (105) reflection (Figure 10e). Concurrently, the P2 (002) reflection also shifts to lower 2θ, confirming the expansion of the c parameter (Figure 10a). In contrast, the P2 (100) and (102) reflections at 2θ° ≈ 16.9° and 18.6°, along with the O3 (101) and (012) reflections at 2θ ≈ 16.7° and 17.5°, shift toward higher angles, signifying a contraction of *a* parameter across all phases (Figure 10a,d). Additionally, the reversible emergence of (110) and ($20\bar{1}$) reflections associated with the O3′ phase, along with (200) and ($11\bar{1}$) reflections characteristic of the P3′ phase, confirms the presence of monoclinic distortions in both O3 and P3 phases. These structural changes are consistent with prior reports [78-81]. Upon discharge, the (00l) reflections characteristic of the O3 phase progressively reappear, verifying the reversible P3 to O3 phase transition via the monoclinic P3′ and O3′ intermediates. Simultaneously, the P2 (100) and (102) peaks revert to their initial positions, with no additional reflections emerging, highlighting the structural reversibility of MFN-512 throughout charge/discharge in the 2.0–4.0 V range.

Post-mortem SEM analysis was performed on MFN-323 and MFN-512 cathodes following cyclability studies to assess morphological changes after 100 cycles at 1 C rate. The post-cycled MFN-323 electrode reveals a stepped surface morphology with evident particle cracking and breaking due to repeated Na^+^ insertion/extraction cycles, as highlighted by red dotted areas and arrow in Supporting Information: Figure S3(a). In contrast, MFN-512 particles maintained their structural integrity and morphology after cycling (Supporting Information: Figure S3b). This observed stability in MFN-512 is attributed to the intergrown P2 and O3 phases, which enhance structural robustness and improve the cycling performance of the cathode.

**FIGURE 11 ∣:**
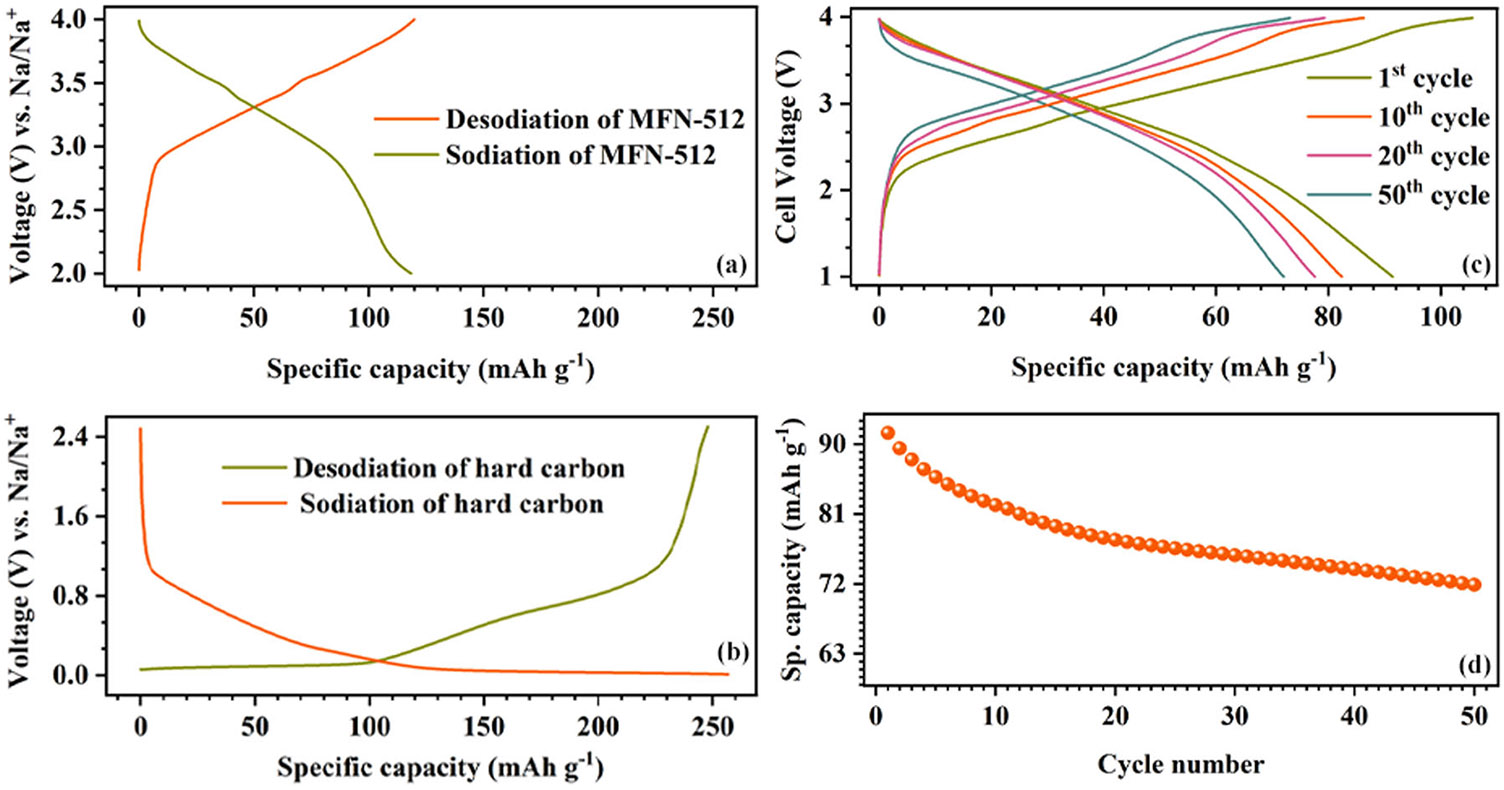
Charge-discharge curves of (a) MFN-512 versus Na metal, (b) hard carbon versus Na metal, and (c) The 1st, 10th, 20th, and 50th cycle GCD curves of full cell with MFN-512 cathode and HC anode at 0.1C. (d) Cyclic performance of MFN-512 – HC full cell at 0.1C for 50 cycles.

To illustrate the practical application of MFN cathodes, a full cell was assembled with MFN-512 as the cathode and hard carbon (HC) as the anode. The hard carbon anode was presodiated in a half-cell setup with sodium metal as the counter electrode. Following this, the sodiated HC was retrieved after de-crimping the coin cell and subsequently integrated into the full cell. The specific capacity of the hard carbon in the second cycle was ~257 mAh g^−1^ (Figure 11b). The full cell was cycled at 0.1C within a voltage window of 1–4 V, achieving a specific capacity of around 92 mAh g^−1^ (calculated based on the active mass of the cathode) with a nominal discharge voltage of ~2.9 V (Figure 11c). After 50 cycles, the full cell retained ~80% of its initial capacity (Figure 11d). It is important to note that the electrochemical performance of the full cell depends on both the cathode and carbon materials. Enhancements in anode stability and kinetics, balancing the active material loadings, and stabilizing the solid electrolyte interphase (SEI) with electrolyte additives could further improve the electrochemical performance of full cells.

## 4 ∣ Conclusions

In summary, a pseudo-ternary Na(Mn-Fe-Ni)O_2_ system with Mn, Fe, and Ni as the axes is explored for layered transition metal oxides as cathodes. A systematic investigation of the influence of varying the fractions of the transition metals on the structural and electrochemical properties is carried out. XRD data revealed that the materials showed a preference for the O3 phase.

- The samples exhibited biphasic behavior in regions with increased Mn content, leading to repulsion between oxygen ions and promoting the formation of prismatic sites. Specifically, MFN-512 displayed O3/P2 phases in a ratio of 35:65, and MFN-521 with a ratio of 17:83 of P2/P3 phases.
- Increasing Ni content causes a decrease in the c parameter, while higher Mn and Fe result in an expanded c parameter.
- Particle sizes generally increased with Fe and Mn content, whereas a higher Ni fraction tends to produce smaller, more agglomerated particles.
- TEM confirmed the coexistence of P2 and O3 phases in MFN-512, contributing to its structural stability and enhanced cyclic performance. Further, superlattice reflections, consistent with the transition metal ordering, were observed in the SAED pattern for the MFN-512 sample. Electrochemical measurements demonstrated that higher Ni content results in greater capacities, with MFN-413 achieving a specific capacity of ~149 mAh g^−1^.
- MFN-422, with a Ni/Fe ratio of 1, demonstrated the best rate performance, with the capacity at 1C being 85.7% of the capacity at 0.1C and at 3C being 69.7% of the capacity at 0.1C. MFN-512 outperformed all samples in terms of cyclic stability, with 93% capacity retention after 100 cycles, which is attributed to the synergistic effect of P2/O3 coexistence. The excellent structural stability of MFN-512 is further confirmed using *operando* Synchrotron XRD which reveals a reversible O3 to P3 phase transition via the monoclinic P3′ and O3′ intermediates during cycling.
- EIS analysis confirms that materials with a higher Mn content along with a lower Fe/Ni ratio have lower charge transfer resistance, which was also reflected by the higher D_Na+_ values of these samples as measured by GITT.
- The practical applicability of MFN-512 was demonstrated in a full cell configuration with hard carbon as the anode, achieving a specific capacity of ~92 mAh g^−1^ with a nominal discharge voltage of ~2.9 V at a 0.1C rate. Overall, MFN-512, with the nominal composition NaMn_0.625_Fe_0.125_-Ni_0.25_O_2_, emerged as the optimal cathode composition among the studied samples due to its high energy efficiency, excellent rate performance, and outstanding cyclability, making it a valuable candidate for next-generation sodium-ion batteries.

## Supplementary Material

Suppliment

## Figures and Tables

**TABLE 1 ∣:** Lattice parameters of the MFN samples obtained from the Rietveld refinement of XRD data.

Sample	Phase	*a* (Å)	c (Å)	*V* (Å^3^)	*R_wp_*, *R_exp_*, *R_p_*, *GOF*
MFN-323	O3	2.97789 ± 0.00004	15.9883 ± 0.0004	122.788 ± 0.005	3.32, 2.33, 2.42, 1.42
MFN-413	O3	2.95129 ± 0.00006	16.0974 ± 0.0005	121.427 ± 0.007	3.34, 2.41, 2.52, 1.39
MFN-332	O3	2.97030 ± 0.00007	16.1269 ± 0.0007	123.221 ± 0.008	2.97, 2.63, 2.36, 1.13
MFN-422	O3	2.94432 ± 0.00005	16.3046 ± 0.0006	122.409 ± 0.006	3.34, 2.63, 2.57, 1.27
MFN-512	O3 35 ± 1	2.92553 ± 0.00007	16.4141 ± 0.0006	121.663 ± 0.008	2.96, 2.07, 2.32, 1.43
	P2 65 ± 1	2.90065 ± 0.00004	11.1194 ± 0.0003	81.023 ± 0.004	
MFN-431	O3	2.9373 ± 0.0002	16.497 ± 0.001	123.27 ± 0.02	2.97, 2.24, 2.23, 1.32
MFN-521	P3 83 ± 2	2.9099 ± 0.0001	16.820 ± 0.002	123.34 ± 0.02	3.26, 3.01, 2.59, 1.08
	P2 17 ± 2	2.9054 ± 0.0009	11.166 ± 0.006	81.63 ± 0.07	

**TABLE 2 ∣:** A comparison of the electrochemical performance of MFN samples.

Sample	First discharge specific capacity (mAh g^−1^)	Average voltage (V)	Energy efficiency at 0.1C(%)	Capacity retention at 1C (of the capacity at 0.1C) (%)	Capacity retention at 3C (of the capacity at 0.1C) (%)	Capacity retention (%) after 100 cycles
MFN-413	160.6	3.07	91.48	81.1	64.8	77
MFN-323	156	3.02	91.81	73.9	47.8	50
MFN-332	120.5	3.00	86.46	75.6	52.9	67
MFN-422	121.8	3.12	89.83	85.7	69.7	80
MFN-512	120.1	3.22	91.35	83.0	59.3	93
MFN-431	60.1	3.04	85.02	66.1	23.2	65
MFN-521	60.2	2.91	86.59	58.8	40.9	75

## Data Availability

Data will be made available on request.
