# Supplementary material for: Probing the Compositional and Structural Effects on the Electrochemical Performance of Na(Mn-Fe-Ni)O2 Cathodes in Sodium-Ion Batteries
Source: Battery Energy. Author manuscript; Available in PMC 2025 Aug 30. (PMC12396823; doi:10.1002/bte2.20240083)
Supplement: Suppliment [file NIHMS2069031-supplement-Suppliment.docx]

**Supporting Information**

**Probing the Compositional and Structural Effects on the Electrochemical Performance of Na(Mn-Fe-Ni)O_2_ Cathodes in Sodium-Ion Batteries**

Samriddhi Saxena^a^, Hari Narayanan Vasavan^a^, Neha Dagar^a^, Karthik Chinnathambi^b^, Asish Kumar Das^a^, Pratiksha Gami^a^, Sonia Deswal^c^, Pradeep Kumar^c^, Sunil Kumar^a,*^

*^a^ Department of Metallurgical Engineering and Materials Science, Indian Institute of Technology Indore, Simrol, 453552, India*

*^b^Micron School of Materials Science and Engineering, Boise State University, Boise, ID 83725, USA*

*^c^ School of Physical Sciences, Indian Institute of Technology Mandi, Mandi, 175005, India*

*^*^E-mail: sunil@iiti.ac.in*

**Tables S1-S7** Crystallographic parameters of MFN samples obtained from Rietveld refinement of room temperature XRD data.

**S1: MFN-323**

| ***O3 (Space Group: R***$\bar{\boldsymbol{3}}$***m)*** | | | | | |
| --- | --- | --- | --- | --- | --- |
| **Atom** | **x** | **y** | **z** | **Occupancy** | **Site** |
| Na1 | 0 | 0 | ½ | 1 | 3b |
| Mn/Fe/Ni | 0 | 0 | 0 | 0.375/0.25/0.375 | 3a |
| O | 0 | 0 | 0.268±0.005 | 1 | 6c |

**S2: MFN-332**

| ***O3 (Space Group: R***$\bar{\boldsymbol{3}}$***m)*** | | | | | |
| --- | --- | --- | --- | --- | --- |
| **Atom** | **x** | **y** | **z** | **Occupancy** | **Site** |
| Na1 | 0 | 0 | ½ | 1 | 3b |
| Mn/Fe/Ni | 0 | 0 | 0 | 0.375/0.375/0.25 | 3a |
| O | 0 | 0 | 0.269±0.003 | 1 | 6c |

**S3: MFN-413**

| ***O3 (Space Group: R***$\bar{\boldsymbol{3}}$***m)*** | | | | | |
| --- | --- | --- | --- | --- | --- |
| **Atom** | **x** | **y** | **z** | **Occupancy** | **Site** |
| Na1 | 0 | 0 | ½ | 1 | 3b |
| Mn/Fe/Ni | 0 | 0 | 0 | 0.5/0.125/0.375 | 3a |
| O | 0 | 0 | 0.270±0.003 | 1 | 6c |

**S4: MFN-422**

| ***O3 (Space Group: R***$\bar{\boldsymbol{3}}$***m)*** | | | | | |
| --- | --- | --- | --- | --- | --- |
| **Atom** | **x** | **y** | **z** | **Occupancy** | **Site** |
| Na1 | 0 | 0 | ½ | 1 | 3b |
| Mn/Fe/Ni | 0 | 0 | 0 | 0.5/0.25/0.25 | 3a |
| O | 0 | 0 | 0.275±0.001 | 1 | 6c |

**S5: MFN-431**

| ***O3 (Space Group: R***$\bar{\boldsymbol{3}}$***m)*** | | | | | |
| --- | --- | --- | --- | --- | --- |
| **Atom** | **x** | **y** | **z** | **Occupancy** | **Site** |
| Na1 | 0 | 0 | ½ | 1 | 3b |
| Mn/Fe/Ni | 0 | 0 | 0 | 0.5/0.375/0.125 | 3a |
| O | 0 | 0 | 0.260±0.001 | 1 | 6c |

**S6: MFN-512**

| ***O3 (Space Group: R***$\bar{\boldsymbol{3}}$***m)*** | | | | | |
| --- | --- | --- | --- | --- | --- |
| **Atom** | **x** | **y** | **z** | **Occupancy** | **Site** |
| Na1 | 0 | 0 | ½ | 1 | 3b |
| Mn/Fe/Ni | 0 | 0 | 0 | 0.625/0.125/0.25 | 3a |
| O | 0 | 0 | 0.277±0.003 | 1 | 6c |
| ***P2 (Space Group : P*6_3_*/mmc)*** | | | | | |
| **Atom** | **x** | **y** | **z** | **Occupancy** | **Site** |
| Na1 | 2/3 | 1/3 | 1/4 |  | 2d |
| Na2 | 0 | 0 | 1/4 |  | 2b |
| Mn/Fe/Ni | 0 | 0 | 0 | 0.625/0.125.0.25 | 2a |
| O | 2/3 | 1/3 | 0.091±0.002 | 1 | 4f |

**S7: MFN-512**

| ***P2 (Space Group : P*6_3_*/mmc)*** | | | | | |
| --- | --- | --- | --- | --- | --- |
| **Atom** | **x** | **y** | **z** | **Occupancy** | **Site** |
| Na1 | 2/3 | 1/3 | 1/4 |  | 2d |
| Na2 | 0 | 0 | 1/4 |  | 2b |
| Mn/Fe/Ni | 0 | 0 | 0 | 0.625/0.25/0.125 | 2a |
| O | 2/3 | 1/3 | 0.092±0.001 | 1 | 4f |
| ***P3 (Space Group: R*3*m)*** | | | | | |
| **Atom** | **x** | **y** | **z** | **Occupancy** | **Site** |
| Na | 0 | 0 | 0.17 | 1.00 | 3a |
| Mn/Fe/Ni | 0 | 0 | 0 | 0.625/0.25/0.125 | 3a |
| O1 | 0 | 0 | 0.38±0.02 | 1.00 | 3a |
| O2 | 0 | 0 | 0.62±0.02 | 1.00 | 3a |

**Table S8.** Transition metal layer and sodium layer spacings in the O3 phase calculated from the refinement data

| ***Sample*** | ***S_TMO2_* (Å)** | ***S_NaO2_* (Å)** |
| --- | --- | --- |
| MFN-431 | 2.42 | 3.08 |
| MFN-323 | 2.09 | 3.24 |
| MFN-332 | 2.07 | 3.30 |
| MFN-413 | 2.04 | 3.33 |
| MFN-422 | 1.90 | 3.53 |
| MFN-512 | 1.85 | 3.62 |

**Table S9.** Comparison of electrochemical properties for various ternary cathode materials with Mn-Fe-Ni for sodium-ion batteries.

| **Composition** | **Phase** | **Specific Capacity (mAh g^-1^)** | **Redox couple responsible for charge compensation** | **Capacity retention at 1C (of the capacity at 0.1C) (%)** | **Cyclability**  **(%, C-rate, Cycles)** |
| --- | --- | --- | --- | --- | --- |
| NaNi_1/3_Mn_1/3_Fe_1/3_O_2_ ^1^ | O3 | 124 (0.2C) | Ni^2+^/Ni^4+^ | 77 | 82%, 1C, 100 |
| NaNi_1/3_Mn_1/3_Fe_1/3_O_2_ ^2^ | O3 | 134 (0.2C) | Ni^2+^/Ni^4+^, Fe^+3^/Fe^+4^ | 78 | 83%, 1C, 100 |
| NaNi_1/3_Mn_1/3_Fe_1/3_O_2_ ^3^ | O3 | 140 (0.1C) | Ni^2+^/Ni^4+^ | 82 | 75%, 1C, 100 |
| NaNi_1/3_Mn_1/3_Fe_1/3_O_2_ ^4^ | O3 | 150 (0.2 C) | Ni^2+^/Ni^4+^, Fe^+3^/Fe^+4^ | 78 | 66%, 2C, 100 |
| NaNi_1/3_Fe_1/3_Mn_1/3_O_2_ ^5^ | O3 | 125 (0.1C) | Ni^2+^/Ni^4+^ | 80 | 88%, 0.5C, 100 |
| NaNi_1/3_Fe_1/3_Mn_1/3_O_2_ ^6^ | O3 | 92 (1C) | Ni^2+^/Ni^4+^, Fe^+3^/Fe^+4^ | 79 | 84%, 1C, 100 |
| NaNi_0.25_Fe_0.25_Mn_0.5_O_2_ ^7^ | O3 | 193 (0.2C) | Ni^2+^/Ni^4+^, Fe^+3^/Fe^+4^ | 83 | 40%, 0.5C, 50 |
| Na[Mn_0.4_Fe_0.3_Ni_0.3_]O_2_ ^8^ | O3 | 167 (0.1C) | Ni^2+^/Ni^4+^, Fe^+3^/Fe^+4^ | 78 | 65%, 0.1C, 100 |
| NaNi_0.60_Fe_0.25_Mn_0.15_O_2_^9^ | O3 | 190 (0.1C) | Ni^2+^/Ni^4+^, Fe^+3^/Fe^+4^ | 75 | 81.3%, 0.2C, 100 |
| Na[Ni_0.55_Mn_0.35_Fe_0.1_]O_2_ ^10^ | P2/O3 | 152 (0.1C) | Ni^2+^/Ni^4+^, Fe^+3^/Fe^+4^ | 65 | 50%, 1C, 100 |
| NaNi_0.4_Mn_0.4_Fe_0.2_O_2_ ^11^ | O3 | 146.2 (0.1C) | Ni^2+^/Ni^4+^, Fe^+3^/Fe^+4^ | 75 | 83%, 1C, 100 |
| **NaMn_0.625_Fe_0.125_Ni_0.250_O_2_ (This work)** | **P2/O3** | **120 (0.1C)** | **Ni^2+^/Ni^4+^** | **83** | **93%, 1C, 100** |

**Table S10.** The range of diffusion coefficients (D_Na+_) of MFN samples.

| Sample | D_Na+_ (cm^2^ s^-1^) |
| --- | --- |
| MFN-323 | 1.09 × 10^-12^ – 1.83 × 10^-10^ |
| MFN-332 | 1.55 × 10^-12^ – 2.50 × 10^-10^ |
| MFN-413 | 4.46 × 10^-12^ – 1.27 × 10^-10^ |
| MFN-422 | 6.17 × 10^-12^ – 5.79 × 10^-10^ |
| MFN-431 | 1.6 × 10^-12^ – 1.89 × 10^-10^ |
| MFN-512 | 8.79 × 10^-12^ – 4.74 × 10^-10^ |
| MFN-521 | 9.94 × 10^-13^ – 3.65 × 10^-10^ |

**Table S11.** The resistance of various components in the MFN cells.

| Sample | Fresh cell (Ω) | | | | Cycled cell (Ω) | | | | % Change in R_Total_ |
| --- | --- | --- | --- | --- | --- | --- | --- | --- | --- |
|  | R_E_ | R_CEI_ | R_CT_ | R_Total_ | R_E_ | R_CEI_ | R_CT_ | R_Total_ |  |
| MFN-323 | 3.8 | 45.9 | 146.9 | 196.6 | 4.1 | 58.7 | 348.1 | 410.9 | 109.2 |
| MFN-332 | 3.5 | 75.2 | 165.0 | 243.7 | 3.9 | 76.4 | 207.1 | 287.8 | 18.1 |
| MFN-413 | 4.0 | 44.5 | 153.9 | 204.4 | 3.9 | 49.2 | 178.9 | 230 | 12.7 |
| MFN-422 | 3.9 | 11.4 | 127.4 | 142.7 | 4.1 | 13.0 | 141.7 | 158.8 | 11.2 |
| MFN-431 | 3.9 | 12.0 | 238.4 | 254.3 | 3.8 | 12.6 | 306 | 322 | 26.6 |
| MFN-512 | 3.7 | 23.4 | 160.0 | 187.1 | 3.7 | 32.4 | 163.3 | 199.4 | 6.6 |
| MFN-521 | 3.8 | 13.6 | 107.2 | 124.6 | 3.7 | 16 | 128 | 147.7 | 18.5 |





**Figure S1.** (a) XRD patterns of the MFN samples. Magnified view of (a1) P2(002), P3(003), and O3(003) peaks, (a2) P2(102) &P2(103), P3(104) & P3(105), and O3(104) & O3(105) peaks.


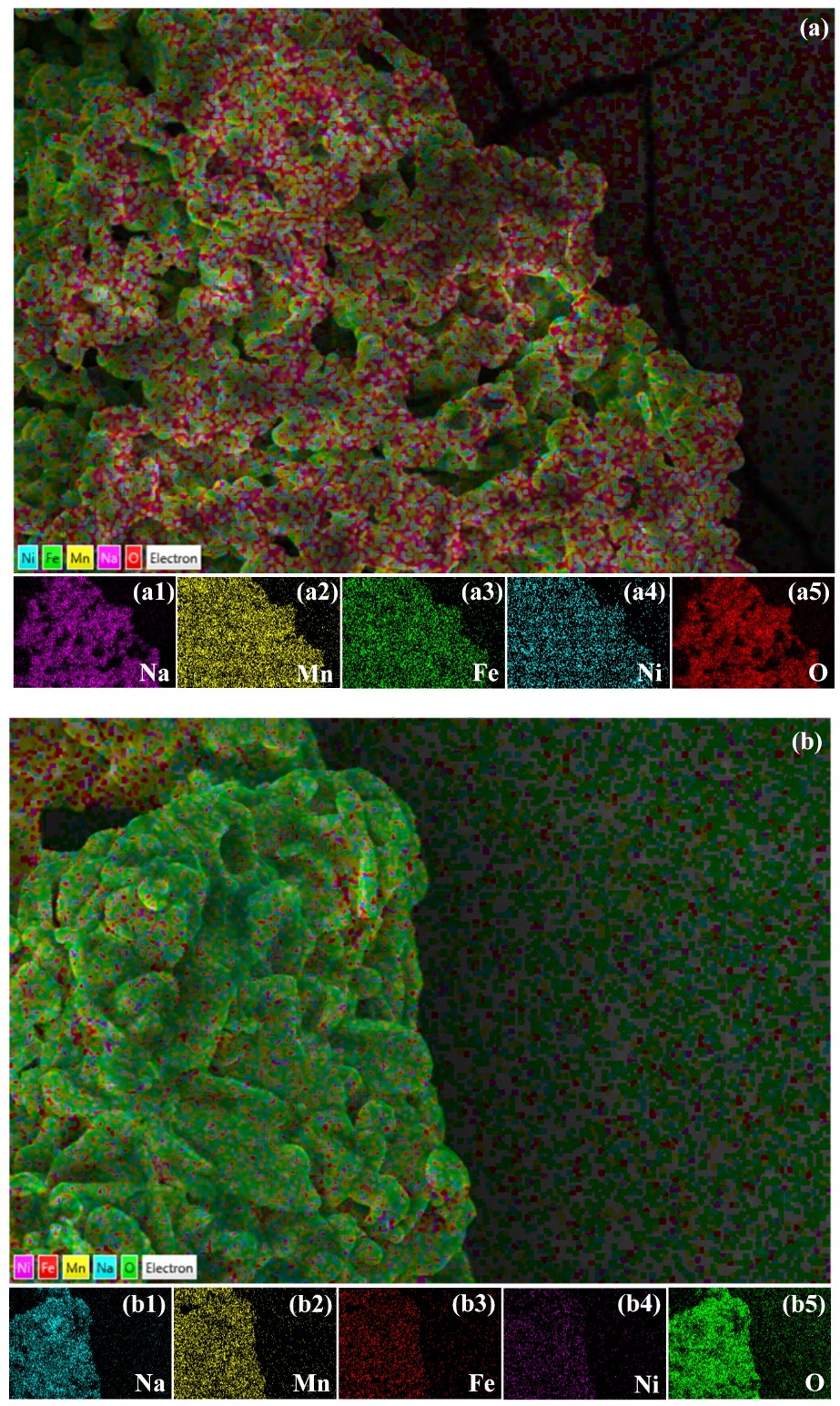


**Figure S2.** EDS elemental mappings of (a) MFN-422 and (b) MFN-512.


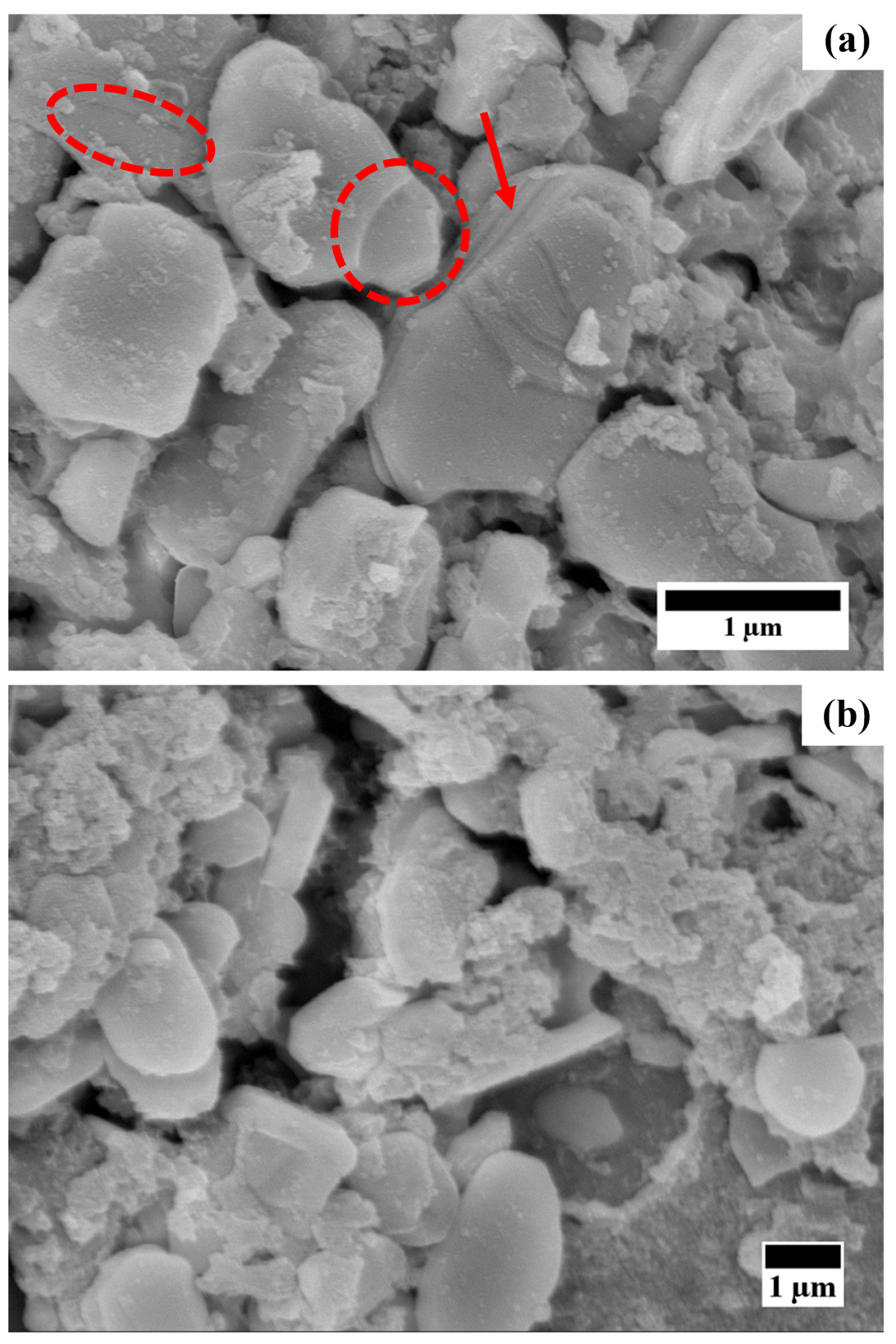


**Figure S3.** SEM images of (a) MFN-323 and (b) MFN-512 electrodes after 100 cycles. The red dotted areas and the arrow indicate the breaking and cracking of MFN-323 particles.

**References**

(1) Qin, W.; Liu, Y.; Liu, J.; Yang, Z.; Liu, Q. Boosting the ionic transport and structural stability of Zn-doped O3-type NaNi_1/3_Mn_1/3_Fe_1/3_O_2_ cathode material for half/full sodium-ion batteries. *Electrochim. Acta* **2022**, *418*, 140357. DOI: 10.1016/j.electacta.2022.140357.

(2) Liu, Q.; Liu, J.; Yang, Z.; Miao, H.; Liu, Y. A high rate and stability cathode material for half/full sodium-ion batteries: Nb-substituted NaNi_1/3_Mn_1/3−x_Fe_1/3_Nb_x_O_2_ layered oxides. *J. Alloys Compd.* **2023**, *968*, 172272. DOI: 10.1016/j.jallcom.2023.172272.

(3) Feng, S.; Zheng, C.; Song, Z.; Wu, X.; Wu, M.; Xu, F.; Wen, Z. Boosting fast ionic transport and stability of O3-NaNi_1/3_Fe_1/3_Mn_1/3_O_2_ cathode via Al/Cu synergistically modulating microstructure for high-rate sodium-ion batteries. *Chem. Eng. J.* **2023**, *475*, 146090. DOI: 10.1016/j.cej.2023.146090.

(4) Li, N.; Wang, S.; Zhao, E.; Yin, W.; Zhang, Z.; Wu, K.; Xu, J.; Kuroiwa, Y.; Hu, Z.; Wang, F.; et al. Tailoring interphase structure to enable high-rate, durable sodium-ion battery cathode. *Journal of Energy Chemistry* **2022**, *68*, 564-571. DOI: 10.1016/j.jechem.2021.12.018.

(5) Deng, C.; Gabriel, E.; Skinner, P.; Lee, S.; Barnes, P.; Ma, C.; Gim, J.; Lau, M. L.; Lee, E.; Xiong, H. Origins of Irreversibility in Layered NaNi_x_Fe_y_Mn_z_O_2_ Cathode Materials for Sodium Ion Batteries. *ACS Appl. Mater. Interfaces* **2020**, *12* (46), 51397-51408. DOI: 10.1021/acsami.0c13850.

(6) Xu, S.; Chen, H.; Li, C.; Nie, R.; Yang, Y.; Zhou, M.; Zhang, X.; Zhou, H. A new high-performance O3-NaNi_0.3_Fe_0.2_Mn_0.5_O_2_ cathode material for sodium-ion batteries. *Ionics* **2023**, *29* (5), 1873-1885. DOI: 10.1007/s11581-023-04963-7.

(7) Hwang, J.-Y.; Myung, S.-T.; Sun, Y.-K. Quaternary Transition Metal Oxide Layered Framework: O3-Type Na[Ni_0.32_Fe_0.13_Co_0.15_Mn_0.40_]O_2_ Cathode Material for High-Performance Sodium-Ion Batteries. *The Journal of Physical Chemistry C* **2018**, *122* (25), 13500-13507. DOI: 10.1021/acs.jpcc.7b12140.

(8) Voronina, N.; Kim, H. J.; Shin, M.; Myung, S.-T. Rational design of Co-free layered cathode material for sodium-ion batteries. *J. Power Sources* **2021**, *514*, 230581. DOI: 10.1016/j.jpowsour.2021.230581.

(9) Ding, F.; Zhao, C.; Zhou, D.; Meng, Q.; Xiao, D.; Zhang, Q.; Niu, Y.; Li, Y.; Rong, X.; Lu, Y.; et al. A Novel Ni-rich O3-Na[Ni_0.60_Fe_0.25_Mn_0.15_]O_2_ Cathode for Na-ion Batteries. *Energy Storage Mater.* **2020**, *30*, 420-430. DOI: 10.1016/j.ensm.2020.05.013.

(10) Mishra, R.; Tiwari, R. K.; Patel, A.; Tiwari, A.; Singh, R. K. A twofold approach for prolonging the lifespan of cobalt-free Na[Ni_0.55_Mn_0.35_Fe_0.1_]O_2_ cathode via Bi^5+^-doping and Bi_2_O_3_ coating in sodium ion batteries. *J. Energy Storage* **2024**, *77*, 110058. DOI: 10.1016/j.est.2023.110058.

(11) Zhao, H.; Li, J.; Liu, W.; Xu, H.; Gao, X.; Shi, J.; Yu, K.; Ding, X. Integrated titanium-substituted air stable O3 sodium layered oxide electrode via a complexant assisted route for high capacity sodium-ion battery. *Electrochim. Acta* **2021**, *388*, 138561. DOI: 10.1016/j.electacta.2021.138561.
